# Optimizing influenza prevention: a systematic review of the cost-effectiveness of pediatric vaccination programs and vaccine types

**DOI:** 10.3389/fpubh.2025.1589403

**Published:** 2025-10-30

**Authors:** Nilay Aksoy, Nur Ozturk, Tamas Agh, Przemyslaw Kardas, Bayram Şahin

**Affiliations:** ^1^Department of Clinical Pharmacy, School of Pharmacy, Altınbaş University, Istanbul, Türkiye; ^2^Clinical Pharmacy PhD Program, Graduate School of Health Sciences, Istanbul Medipol University, Istanbul, Türkiye; ^3^Syreon Research Institute, Budapest, Hungary; ^4^Medication Adherence Research Group, Center for Health Technology Assessment and Pharmacoeconomic Research, University of Pecs, Pecs, Hungary; ^5^Medication Adherence Research Centre, Department of Family Medicine, Medical University of Lodz, Lodz, Poland; ^6^Faculty of Economics and Administrative Sciences, Department of Health Care Management, Hacettepe University, Ankara, Türkiye; ^7^Health Sciences Institute, Department of Health Management, Lokman Hekim University, Ankara, Türkiye

**Keywords:** pediatric influenza vaccination, cost-effectiveness, pharmacoeconomics, quadrivalent vaccine, live-attenuated vaccine

## Abstract

**Introduction:**

Seasonal influenza is a major cause of illness and death worldwide. Vaccination remains the cornerstone of prevention, with options including trivalent inactivated (TIV), quadrivalent inactivated (QIV), and live-attenuated vaccines. This study aimed to provide a systematic overview of the cost-effectiveness of pediatric influenza vaccination programs, with a particular focus on comparing different vaccine types.

**Methods:**

A comprehensive literature search was conducted in PubMed, Web of Science, Scopus, and Cochrane databases for records published between 2013 and 2024. The target population included individuals younger than 18 years. The primary research question was: Which influenza vaccines, trivalent, quadrivalent, or live-attenuated, are more cost-effective, and how does introducing seasonal vaccination for children under 18 influence healthcare costs and health outcomes? Data extraction was performed using a structured Excel spreadsheet.

**Results:**

This review included 33 studies that met the inclusion and exclusion criteria. Most studies support the conclusion that vaccinating children is an effective and cost-effective strategy for reducing influenza transmission. Cost-effectiveness varied depending on epidemiological and demographic factors, the type of vaccine used, and age group differences, which were influenced by the analytical perspective and local health and economic conditions.

**Conclusion:**

This review confirms that pediatric influenza vaccination is a cost-effective intervention, particularly with quadrivalent vaccines. The optimal choice of vaccine and strategy should be tailored to local population needs and economic conditions to maximize public health benefits.

## 1 Introduction

Seasonal influenza is a major contributor to severe illness and hospitalization. Globally, it affects approximately one billion individuals each year, including 3–5 million cases that are life-threatening. These cases result in an estimated 290,000 to 650,000 deaths annually, with most fatalities in children under five attributed to influenza-related lower respiratory tract infections (1).

Vaccination remains the most effective strategy for preventing infection and reducing the severe consequences of seasonal influenza (2).

Most inactivated vaccines are produced in embryonated hen eggs, although some now use mammalian cell lines. The monovalent antigens are combined into trivalent or quadrivalent formulations. Trivalent influenza vaccines (TIVs) include two influenza A strains (H1N1 and H3N2) and one influenza B strain, whereas quadrivalent influenza vaccines (QIVs) include both lineages of influenza B (3). QIVs were developed to address the challenge of accurately identifying the predominant B lineage for TIV (4).

Annual influenza immunization is recommended due to the yearly variation in circulating virus strains, corresponding changes in vaccine composition, and the gradual decline in immunity over time. Ritzwoller et al. (5) reported that, although the influenza vaccine did not perfectly match the predominant circulating strains, it provided substantial protection to children who received the full dose and partial protection to those who received only one dose and were younger than nine years.

These findings highlight the importance of administering two doses to previously unvaccinated children for optimal protection and support vaccinating eligible children even when the vaccine strain match is suboptimal (5).

The live attenuated intranasal vaccine (LAIV), introduced in 2003, is also produced in embryonated hen eggs and does not contain preservatives. Like inactivated vaccines, it is updated annually and is recommended for individuals who are healthy, not pregnant, and between 2 and 49 years of age.

Since 2012, the World Health Organization (WHO) has recommended influenza vaccination for children aged 6 to 59 months, pregnant women, older adults, individuals with chronic illnesses, and healthcare professionals (6). Finland, the United Kingdom (UK), and Canada have incorporated the pediatric population into their routine immunization schedules, while the United States has adopted a universal vaccination policy covering the entire population from 6 months of age (7, 8).

In most European countries, vaccination is advised for individuals aged 65 years and older; however, the age threshold has been lowered to 60 years in Italy, Germany, Greece, Iceland, Hungary, and the Netherlands (9). Similarly, in Türkiye, annual influenza vaccination is recommended for people aged 65 years and older, children aged six months and above, individuals with chronic illnesses, nursing home residents, pregnant women, and healthcare personnel. The cost of vaccination for these high-risk groups is fully covered by the healthcare system (10).

Although substantial evidence supports the effectiveness of vaccines in children under five, limited data are available for those aged 5 to 14 years and young adults up to 18 years. While influenza often has less severe consequences in this age group, recent research indicates that the average age of influenza-related deaths among children without pre-existing conditions is approximately 6 to 7 years (11).

Administering influenza vaccines throughout childhood is an effective strategy for reducing the overall burden of the disease, particularly by lowering rates of hospitalization and complications in children. Vaccinating children also helps curb transmission to more vulnerable individuals, such as those with weaker immune responses and older adults (12).

Economic evaluations have demonstrated that vaccinating children significantly decreases the incidence of influenza, leading to substantial reductions in healthcare costs (13). These savings stem from fewer physician consultations, emergency room visits, hospitalizations, and prescriptions. Widespread vaccination also reduces school absenteeism, supports better educational outcomes, and helps parents maintain consistent work schedules. Importantly, pediatric immunization contributes to the establishment of herd immunity, providing indirect protection to susceptible populations and further reducing the economic and healthcare burden associated with influenza (14, 15). While evidence supports the general benefits of pediatric influenza vaccination, studies specifically comparing the cost-effectiveness of different vaccine types are limited. This systematic review addresses this gap by providing an overview of the cost-effectiveness of pediatric vaccination programs, with a particular focus on differences across vaccine types.

## 2 Methods

We systematically searched four electronic databases, namely PubMed, Scopus, Web of Science, and Cochrane, for records published between 2013 and 2024. The target population consisted of individuals younger than 18 years. The study aimed to answer the following question: Which influenza vaccines are more cost-effective—trivalent, quadrivalent, or live-attenuated—and how does the introduction of seasonal vaccination for children under 18 affect healthcare costs and health outcomes?

**Figure 1:**
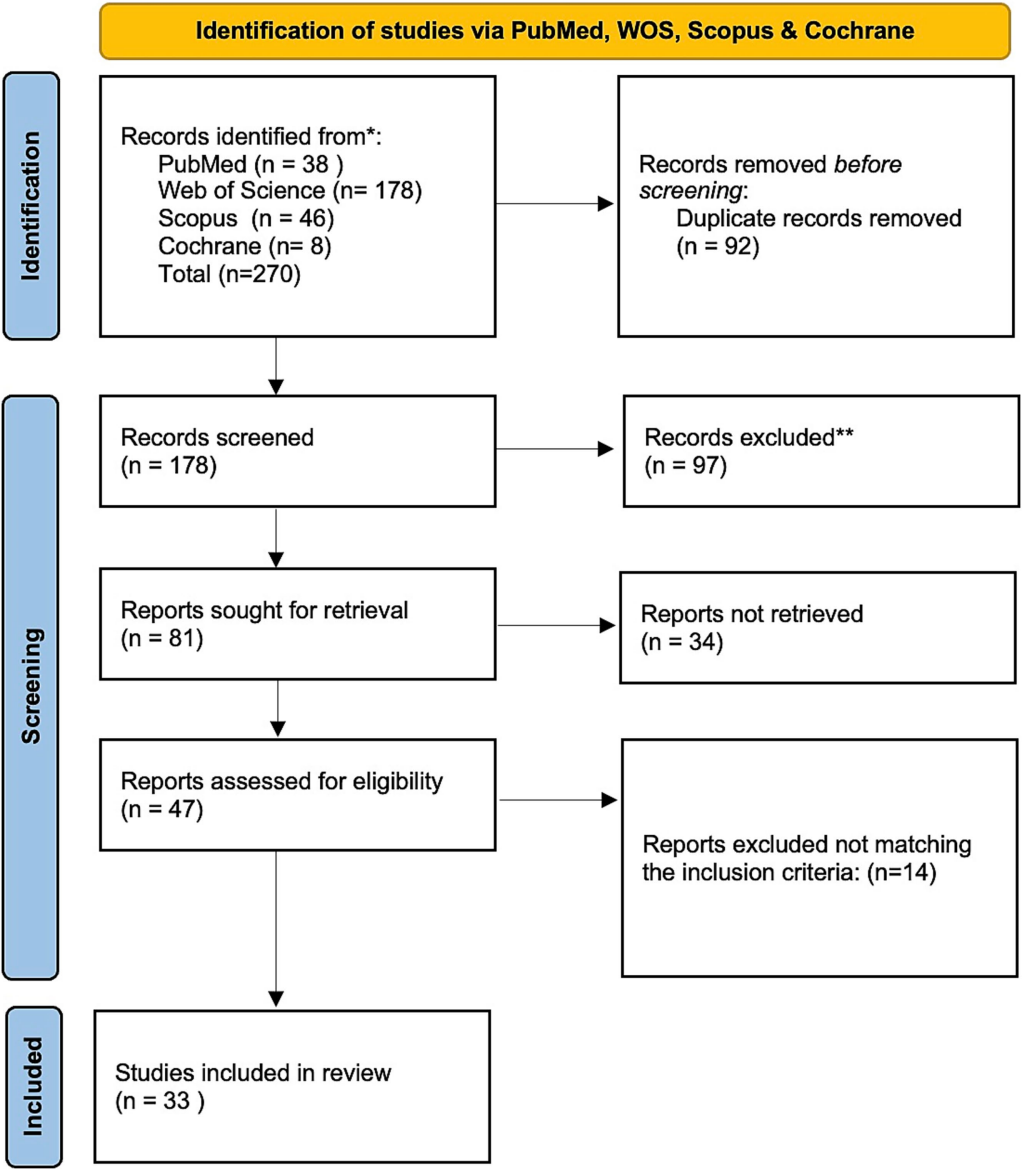
PRISMA flow chart for the studies included in the systematic review.

This review followed the Preferred Reporting Items for Systematic Reviews and Meta-Analyses (PRISMA) guidelines, using a flow diagram to map the literature selection process (16) (Figure 1). The search string combined the following keywords: “((pharmaeconomic OR pharmaceutical economics OR pharmaceutical economy OR pharmacy economy OR cost effectiveness OR economic OR cost OR expenditure OR value AND money OR budget) AND ((vaccine OR immune* OR active immunization*)) AND ((flu OR influenza* OR human influenza* OR human flu OR influenza in human* OR grippe OR seasonal flu)) AND ((pediatric* OR child OR child health OR healthy children* OR children’s health OR child wellbeing OR children*)).”

### 2.1 Inclusion and exclusion criteria

The inclusion criteria encompassed clinical research evaluating the cost-effectiveness of seasonal influenza vaccines in children under 18 years of age. No restrictions were applied regarding country of origin, participant race or gender, or the language of publication. All vaccine formulations, including trivalent, multivalent, and LAIV, were considered. Exclusion criteria included abstract-only papers, conference proceedings, editorials, author responses, theses, books, articles without full-text availability, case reports, case series, systematic reviews, meta-analyses, and studies comparing antiviral treatments. In addition, studies that did not clearly describe the cost-analysis methodology or that lacked data on incremental cost-effectiveness were excluded.

### 2.2 Screening and data retrieval

Search hits were first de-duplicated to account for overlap between databases, followed by double-blinded title, abstract, and full-text screening conducted independently by two reviewers (N.A. and N.O.). Disagreements were resolved by B.S.

A Microsoft Excel spreadsheet was developed to systematically collect and extract data. Two authors (N.A. and N.O.) independently collected and verified the extracted information, which included the following parameters: authors, year of publication, study setting, type of economic evaluation, mathematical model, time horizon, target population, vaccination strategy, analytical perspective, vaccine type and cost, cost drivers, sensitivity analyses, clinical outcomes, incremental cost-effectiveness ratio (ICER), and key study conclusions.

## 3 Results

The literature search identified 270 studies: 38 from PubMed, 178 from Web of Science, 46 from Scopus, and 8 from Cochrane. After removing 92 duplicates, 178 articles remained for title and abstract screening, of which 97 were excluded as irrelevant. The full texts of the remaining 81 publications were reviewed in detail, resulting in 33 articles that met the inclusion and exclusion criteria (Figure 1). Data from these articles were extracted systematically. The PICOS framework (Population, Intervention, Comparisons, Outcomes, and Study design) was used to guide data extraction. Details on population, intervention, comparisons, and study setting and design are presented in Supplementary Table 1, while outcomes related to effectiveness, cost, and ICERs are summarized in Supplementary Tables 2, 3.

These 33 studies evaluated the pharmacoeconomic impact of pediatric influenza vaccination in diverse settings, including countries in Europe (Finland, France, Germany, Ireland, the Netherlands, Portugal, Scotland, Spain, and England), as well as South Korea, Canada, Peru, Uruguay, Bangladesh, China, the United States, South Africa, Argentina, Taiwan, Thailand, and Vietnam.

**Figure 2:**
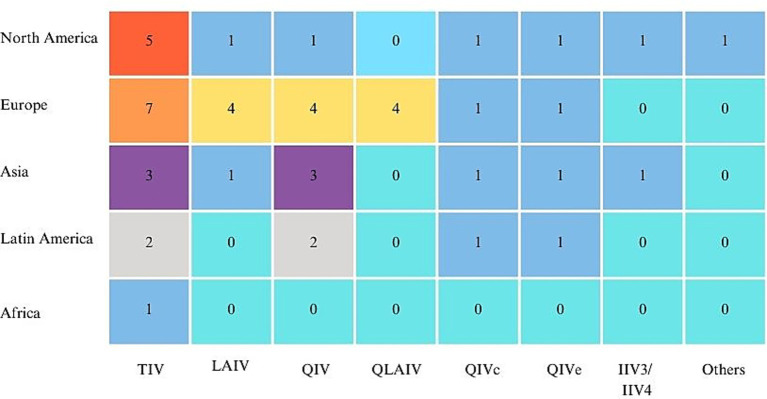
Distribution of vaccine types across different regions.

**Figure 3:**
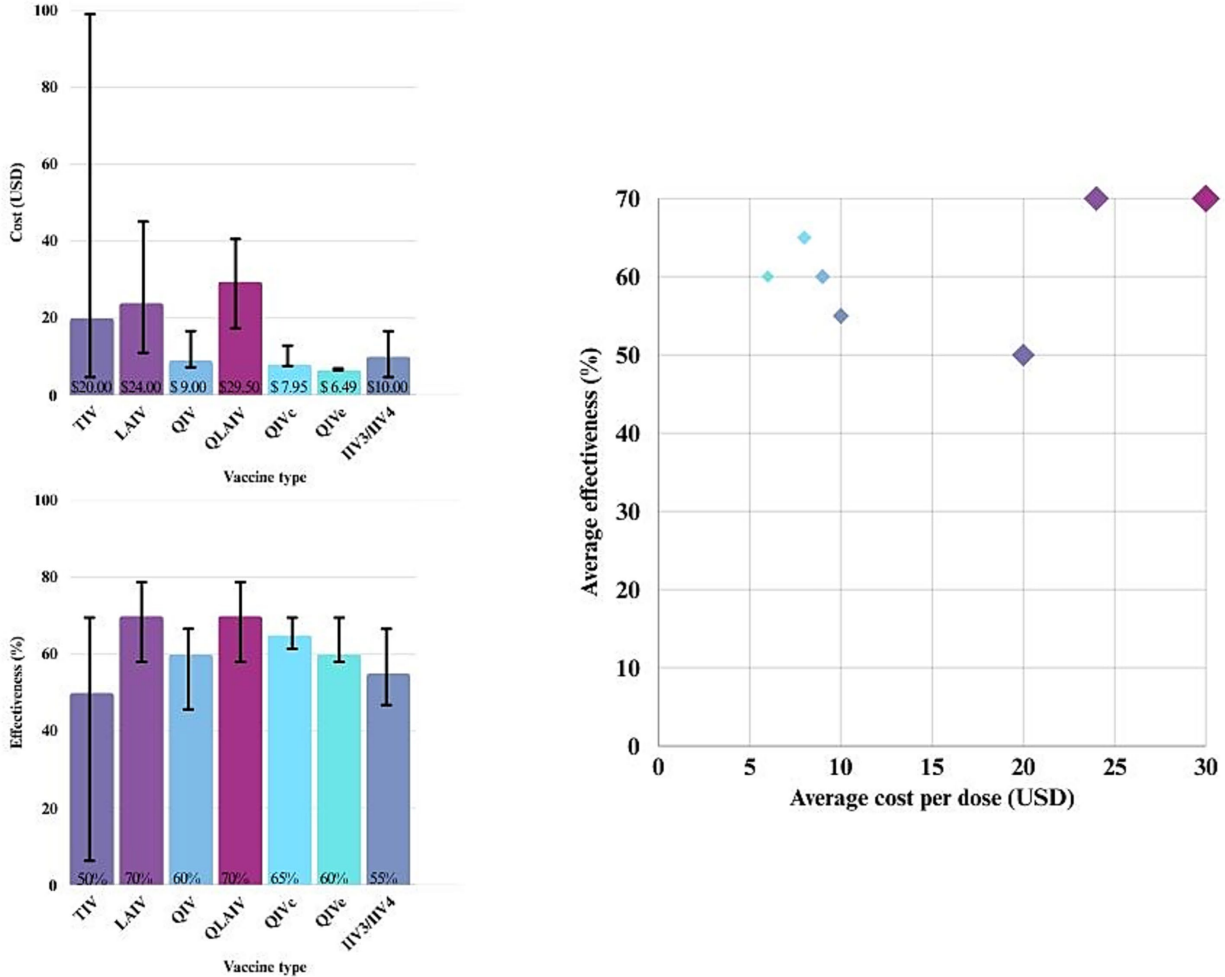
Cost, effectiveness, and coverage of different influenza vaccine types.

Figure 2 illustrates the distribution of studies by region and vaccine type, while Figure 3 presents a scatter plot showing the cost, effectiveness, and coverage of different vaccine types.

Most cost-effectiveness studies used static models, whereas 14 studies employed dynamic transmission models (17–30) (Supplementary Table 1).

The analysis time horizon varied considerably across studies, ranging from 200 years in one study (25) to 20 years in three studies (20, 23). Two studies used a 14-year horizon (17, 29), while three others adopted a 10-year timeframe (19, 22, 28). The remaining studies focused on a single influenza season or a one-year period (31–46) (Supplementary Table 1).

**Figure 4:**
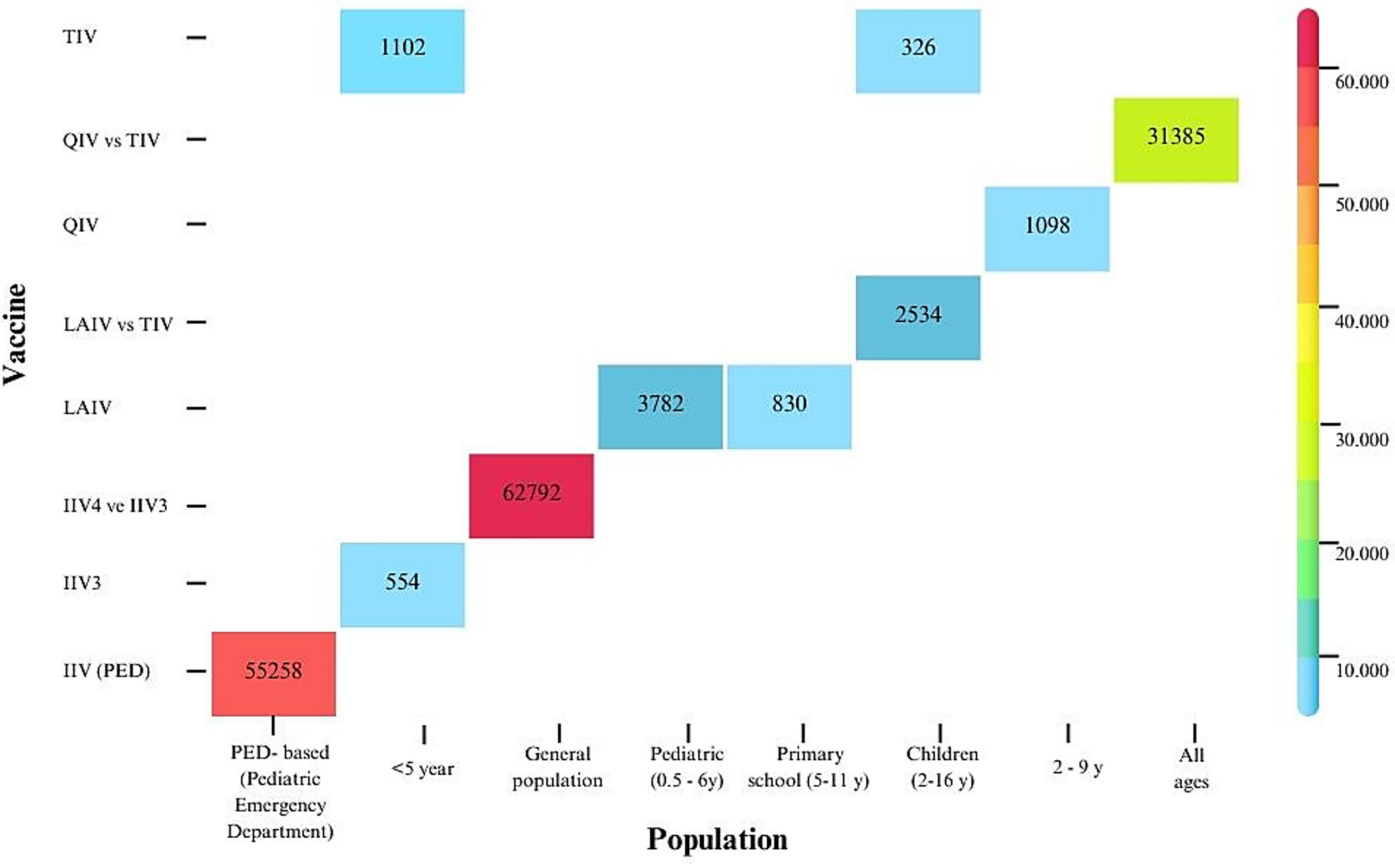
Cost-effectiveness of different influenza vaccine types across age groups.

The populations analyzed also varied widely, ranging from 6 to 56 months (38), 36 months to 15 years (47), and 0 to 17 years (40), to 2 to 16 years (20), and 2 to 11 years or 2 to 16 years in an expanded scenario (29). Pitman et al. (25) assessed three alternative vaccination strategies targeting children aged 2–4, 2–10, and 2–18 years, while Baguelin et al. (17) examined strategies for children aged 2–4, 5–16, and 2–16 years. Figure 4 presents the ICER in USD per quality-adjusted life-year (QALY) for different vaccine comparisons across these age groups.

The cost-effectiveness of different influenza vaccines was evaluated across multiple studies. Seven studies compared QIV with TIV (19, 21, 23, 31, 33, 38, 48). Three studies assessed TIV versus no vaccination (39, 40, 49), whereas two compared QIV with no vaccination (18, 42). Three studies analyzed live-attenuated influenza vaccine (LAIV) in comparison with TIV (17, 19, 25). Thommes et al. (28) evaluated TIV versus QIV in Canada, whereas in the United Kingdom (UK), children aged 2–17 years transitioned from LAIV to quadrivalent live-attenuated influenza vaccine (QLAIV), and adults switched from TIV to QIV. Nagy et al. (23) compared TIV, QIV, and LAIV in pediatric populations and evaluated QIV in other age groups under various scenarios, including no vaccination. All studies conducted sensitivity analyses, with several incorporating multi-parameter sensitivity analyses.

Except for Yoo et al. (46), Gregg et al. (47), and Gerlier et al. (22), which reported outcomes as cost per life-year gained or cost per influenza case averted, nearly all studies used cost per QALY as the primary economic outcome. Detailed results and conclusions are presented in Supplementary Tables 1–3.

### 3.1 Cost-effectiveness of pediatric influenza vaccination programs

Numerous studies (17, 19, 22, 24, 25–29, 31, 32, 34, 36, 37, 44) have confirmed that vaccinating children is both effective and cost-effective in reducing influenza transmission. Edoka et al. (49) reported that vaccinating all at-risk populations, with the exception of children aged 6–59 months, is cost-effective. In contrast, Naber et al. (40) found that annual vaccination of children with medical risk factors is not cost-effective. de Boer et al. (20) concluded that pediatric vaccination is cost-effective in the Netherlands but less so when the analysis is limited to outcomes in children alone. Age-stratified analyses revealed clear differences in cost-effectiveness. Vaccination of primary school-aged children (approximately 5–11 years) consistently demonstrated the most favorable cost-effectiveness profiles, reflecting their central role in influenza transmission and social contact patterns (17, 30). Preschool children (2–4 years) also benefited, with generally cost-saving outcomes, although ICERs were typically higher compared with primary school-aged children, likely due to differences in contact rates and vaccine uptake (25, 30). In contrast, vaccination of secondary school-aged children (12–16 years) yielded mixed results and, in some scenarios, may not be cost-effective (30). Figure 4 illustrates these age-related differences in cost-effectiveness.

### 3.2 Herd immunity, mathematical method, and time horizon

Herd immunity provides indirect protection and is a key driver of the cost-effectiveness of pediatric vaccination (22, 26, 50). Studies such as Baguelin et al. (17), Pitman et al. (25), and Sandmann et al. (26) demonstrate that incorporating herd protection through dynamic modeling reduces ICERs by approximately 10–30%, reflecting the broader population-level benefits of vaccination. In contrast, static decision-tree or Markov models, as used by Yoo et al. (46) and Kittikraisak et al. (39), often generate higher ICERs and may underestimate the full economic value of pediatric vaccination programs. Studies employing longer time horizons, spanning multiple influenza seasons or even several decades [e.g., Baguelin et al. (17), Pitman et al. (25), and Sandmann et al. (26)]—are better able to capture the cumulative benefits of vaccination, including sustained herd immunity and reductions in influenza-related complications over time. These extended horizons typically yield more favorable ICERs by accounting for long-term health gains and cost savings. Conversely, evaluations with shorter horizons, such as single-season static models [e.g., Kittikraisak et al. (39) and Yoo et al. (46)], often underestimate the overall economic value of vaccination by focusing solely on immediate, direct effects.

### 3.3 Perspective of the economic analysis

Cost-effectiveness analyses of pediatric influenza vaccination commonly adopt different perspectives, which significantly influence the reported outcomes.

The societal perspective is frequently used as it captures a comprehensive range of costs and benefits, including direct medical costs, non-medical costs, and indirect costs such as productivity losses due to parental caregiving or absenteeism. This broader viewpoint often results in more favorable cost-effectiveness ratios compared with analyses adopting a healthcare payer perspective, which typically considers only direct healthcare costs borne by insurers or health systems. Our review found that the societal perspective predominates in many studies, particularly in high-income countries, because it better reflects the full economic impact of vaccination programs. Several models and economic analyses have also demonstrated that clinical effects of vaccination, including reductions in illness, hospitalizations, and fatalities, outweigh the cost of vaccination.

For example, A study by Scholz et al. (27) in Germany found that systematic childhood vaccination is not only cost-saving from a societal perspective but also highly cost-effective from the perspective of third-party payers. Similarly, an analysis by Pitman et al. (25), a vaccination strategy targeting children aged 2–18 years in the UK was identified as the most cost-effective, with an ICER of £251 per QALY from the perspective of the National Health Service.

### 3.4 Regional differences

Regions with higher influenza burden often report more favorable ICERs, sometimes even achieving cost-saving outcomes, as vaccination reduces both direct medical expenses and broader societal impacts such as productivity losses (17, 43).

Conversely, in settings with higher vaccine costs or lower influenza incidence, as observed in Uruguay, ICERs may rise and approach or even exceed commonly accepted willingness-to-pay thresholds, potentially challenging the economic viability of vaccination programs (48).

### 3.5 Vaccine efficacy/effectiveness and coverage

The scatter plot in Figure 3 shows that as effectiveness increases, costs generally rise as well. Some strategies cluster in the lower-cost, higher-effectiveness range, while others are associated with higher costs but only modest gains in effectiveness.

Several studies in this systematic review focused on vaccine efficacy in preventing severe influenza-related outcomes, such as hospitalizations and complications, which have a substantial impact on healthcare costs and patient well-being [e.g., Naber et al. (40)]. These studies highlight that reducing severe cases significantly enhances the cost-effectiveness of vaccination programs by preventing expensive medical treatments and improving quality of life. Other studies, such as De Boer et al. (20), emphasize the importance of vaccination coverage, the proportion of the target population that receives the vaccine, as a critical factor influencing cost-effectiveness. Higher coverage rates strengthen herd immunity, reduce disease transmission, and amplify both direct and indirect benefits, thereby improving overall economic outcomes.

Together, these findings underscore that both vaccine effectiveness against severe disease and the extent of vaccine uptake are key drivers in determining the value of pediatric influenza vaccination programs (20, 40).

Live-attenuated vaccines (LAIV and QLAIV) show the highest effectiveness (~70%) but also the highest cost per dose (approximately $24–$29). Quadrivalent inactivated vaccines (QIVc and QIVe) offer a balance of moderate cost ($6.49–$10) and good effectiveness (~60–65%). Among these, egg-based quadrivalent vaccines (QIVe) are slightly less effective than cell-based quadrivalent vaccines (QIVc) due to egg-adaptive mutations that can reduce the match to circulating strains, thereby impacting overall effectiveness (41). Trivalent vaccines (TIV and IIV3/IV4) are less expensive but typically less effective (~50–55%).

Vaccine coverage assumptions in economic models vary widely across studies and populations. For example, Baguelin et al. (17) assumed approximately 20% coverage in children aged 1–14 years and 50% in low-risk groups, whereas Kittikraisak et al. (39) reported coverage ranging from 24 to 64% in different seasons for children and 29 to 31% for adults. Vo et al. (43) modeled a base-case coverage of 97.2%, reflecting near-universal vaccination in their setting. Gerlier et al. (22) simulated an expansion of coverage among healthy children aged 2–17 years to 50%, while Bellier et al. (31) reported coverage ranging from 7% in adults to over 52% in one-year-old children, highlighting age-related differences. Wong et al. (45) observed a baseline intramuscular vaccine acceptance rate of 28.4%, with potential increases through novel delivery methods.

### 3.6 Quadrivalent versus trivalent vaccines

Kim et al. (38) reported that QIV was cost-effective in older populations but not in children aged 6 to 59 months. In contrast, several other studies have shown that QIVs generally provide greater cost-effectiveness than TIVs across a range of age groups. These findings have been consistent across diverse geographic regions and healthcare systems, supporting the broad applicability of QIV (18, 21, 23, 26, 28, 29, 31, 33, 34, 38, 48).

### 3.7 Inactivated versus live-attenuated vaccines

LAIV generally demonstrates higher or comparable effectiveness in children, particularly in younger age groups (2–17 years), compared with inactivated influenza vaccines (22). Several studies (e.g., (17, 25, 29)) have found LAIV to be more effective in preventing influenza in healthy children, likely due to its intranasal administration and ability to induce mucosal immunity. LAIV is also well tolerated and often preferred by children because of its needle-free delivery, which can enhance vaccine uptake (45). Multiple studies (e.g., (19, 22, 46)) have shown that pediatric vaccination programs using LAIV can be cost-effective, and in some cases cost-saving, compared with IIV, due to higher vaccine effectiveness and the added benefits of herd immunity.

### 3.8 Cell-based versus egg-based quadrivalent influenza vaccines

Three studies compared QIVc with QIVe (24, 32, 42). All reported that QIVc generally provides greater vaccine effectiveness than QIVe. Although QIVc has a higher unit cost, it is often found to be cost-effective, and in some cases cost-saving (24), when the broader healthcare costs avoided through better protection are considered.

### 3.9 Economic and health system context

Thommes et al. (28) reported that QIV was more cost-effective in Canada than in the United Kingdom, largely due to higher healthcare expenses and greater vaccination uptake in Canada. Influenza vaccination tends to be more cost-effective in countries with higher healthcare costs and higher vaccination coverage rates. Studies from diverse settings, such as Bangladesh (36) and Taiwan (32), highlight that local healthcare access and economic conditions play a critical role in determining the value and feasibility of vaccination strategies.

## 4 Discussion

This systematic review synthesized evidence from 33 studies evaluating the cost-effectiveness of pediatric influenza vaccination programs across diverse global settings. The findings consistently demonstrate that vaccinating children, particularly primary school-aged groups, is a cost-effective strategy for reducing influenza transmission and its associated health and economic burdens. Importantly, the review highlights key nuances related to vaccine type, regional context, modeling approaches, and age-specific patterns in cost-effectiveness, supporting the prioritization of pediatric vaccination in public health policy.

These results are consistent with previous systematic reviews and economic evaluations, reinforcing that childhood influenza vaccination is generally a cost-effective intervention. Notably, prioritizing primary school-aged children maximizes both direct and indirect benefits, making this group the most economically advantageous target for influenza vaccination programs (51, 52).

A key finding of this review is the substantial impact of herd immunity on the cost-effectiveness of pediatric influenza vaccination. Most studies that incorporated dynamic transmission models capable of capturing indirect protection reported that including herd immunity effects significantly improved cost-effectiveness estimates, often reducing ICERs by 10–30%. For example, studies such as Pitman et al. (25), Baguelin et al. (17), and Sandmann et al. (26) found that accounting for herd immunity led to more favorable economic outcomes compared to static models, which only consider direct effects. This aligns with previous research and systematic reviews [e.g., Boccalini et al. (53)] that emphasize herd immunity as a critical driver of vaccination value. Our review confirms that dynamic models, which reflect real-world transmission dynamics, provide a more accurate assessment of the full benefits of pediatric vaccination programs. However, the magnitude of the herd immunity effect varied depending on local epidemiology, vaccine coverage, and population structure, underscoring the importance of context-specific modeling. Modeling studies of influenza vaccines, along with ecological evidence demonstrating the benefits of herd immunity, have informed seasonal and pandemic influenza vaccination strategies (54, 55). Herd immunity reduces the number of influenza cases among unvaccinated individuals, thereby lowering healthcare expenditures and improving overall health outcomes (56). Earlier simulation studies in the United States have consistently reported strong indirect effects, with even relatively low vaccine coverage rates yielding significant public health benefits (57, 58).

Notably, one study comparing dynamic and static modeling approaches found that the indirect protection conferred to unvaccinated individuals (the “herd immunity effect”) can exceed the direct benefits experienced by vaccine recipients, highlighting the importance of incorporating herd immunity into economic evaluations of preventive interventions (59). Thus, dynamic transmission models are essential for accurately assessing the population-level benefits of pediatric influenza vaccination.

Our review found that the societal perspective predominates in many studies, particularly in high-income countries, because it better captures the full economic impact of vaccination programs. A systematic review of the cost-effectiveness of seasonal influenza vaccines in older adults demonstrated that the chosen perspective significantly influences results. Analyses adopting a healthcare payer viewpoint typically include only direct medical costs, often producing more conservative estimates of vaccine cost-effectiveness. In contrast, societal perspective analyses incorporate indirect costs such as productivity losses and caregiving burdens, generally yielding more favorable cost-effectiveness ratios (60), a finding consistent with the results of this review. Influenza in children frequently leads to parental work absenteeism, imposing substantial economic burdens on families and employers (57). Moreover, adopting a societal perspective supports more equitable health policy decisions by acknowledging the broader social value of vaccination, including reduced disease transmission in underserved communities. This consideration is particularly relevant in low- and middle-income countries, where indirect benefits and productivity gains are key drivers of vaccination value (61). Additionally, employing longer time horizons in conjunction with dynamic transmission models enables a more comprehensive assessment of vaccination programs by capturing indirect and long-term benefits. In contrast, shorter time horizons, while useful for rapid decision-making, risk overlooking these critical effects.

The cost-effectiveness of influenza vaccination programs can vary considerably depending on the economic and healthcare context of a region. For example, countries with higher healthcare costs, such as greater expenses related to hospitalizations, medical treatments, and management of influenza complications, tend to report more favorable cost-effectiveness outcomes, as preventing illness offsets these substantial costs. Higher vaccination uptake rates in such settings further enhance cost-effectiveness by increasing overall health benefits and reducing disease transmission. Conversely, in regions where healthcare costs are lower or influenza incidence is less severe, the economic value of vaccination programs may appear less favorable. Differences in healthcare infrastructure, access to medical services, and local economic conditions also shape the performance of vaccination strategies in terms of both health outcomes and costs. Policymakers should therefore interpret ICERs within their regional context, considering not only economic thresholds but also public health priorities and budget constraints, to optimize vaccination strategies effectively.

Previous studies conducted outside the timeframe of this systematic review have suggested that vaccinating low-risk children may not always be cost-effective. This is particularly evident in regions with low influenza prevalence, where fewer cases occur and, consequently, fewer illnesses are prevented through vaccination. Additionally, when vaccine effectiveness is suboptimal, due to factors such as poor strain match or lower immune response, the health benefits gained from vaccination are reduced. In such contexts, the costs of vaccinating large numbers of low-risk children may outweigh the economic and health benefits, making vaccination programs less economically attractive (15, 57). Variations in healthcare utilization patterns, such as hospitalization rates, outpatient visits, and willingness-to-pay thresholds, also influence cost-effectiveness outcomes (62). Willingness to pay reflects the maximum amount a society or healthcare system is prepared to invest to gain one unit of health benefit (e.g., a QALY), and higher thresholds can make vaccination programs appear more cost-effective by accepting higher costs for greater health gains. These disparities underscore the need for region-specific economic evaluations that incorporate local cost data, vaccine procurement mechanisms, and demographic factors to accurately assess the value of pediatric influenza vaccination.

The findings of this systematic review highlight important considerations for influenza vaccine selection in public health programs, where higher-cost vaccines may provide superior protection, but budget constraints often require selecting vaccines that balance cost and effectiveness. Vaccine coverage assumptions also depend heavily on local context, age groups, and program strategies, which critically affect the projected impact and cost-effectiveness of influenza vaccination. In this review, QIVs demonstrated superior cost-effectiveness across multiple studies.

QIVs provide protection against four influenza virus strains, including two distinct lineages of influenza B viruses. In comparison, TIVs protect against only one B strain. By covering both B lineages, QIVs offer broader protection and reduce the likelihood of a strain mismatch during the influenza season. This advantage is particularly notable in years when influenza B viruses predominate. However, variations in assumptions about vaccine effectiveness, strain circulation, and healthcare utilization contribute to differences in estimated cost-effectiveness across studies. Additionally, differences in vaccination coverage rates and population immunity can influence the incremental benefits of QIV, with some models showing smaller gains in settings with high baseline vaccination coverage. A systematic review of health economic evaluations found that switching from TIV to QIV is generally a valuable intervention from both public health and economic perspectives (63). These findings support our results and underscore that vaccine selection is a key determinant of program value.

The decision between inactivated and live-attenuated vaccinations depends on patient age, risk profile, and local epidemiology. Consistent with our findings, systematic reviews and economic analyses have shown that vaccinating children with the intranasal LAIV is cost-effective compared with strategies targeting only high-risk groups or the older adults, and often when compared with IIV/TIV as well (64–66).

The emergence of QIVc as a potentially more effective, though more expensive, alternative was also examined in this systematic review. Across all included studies, QIVc consistently demonstrated superior vaccine effectiveness compared with QIVe. This advantage is largely attributed to the avoidance of egg-adaptive mutations during vaccine production, which can reduce the antigenic match between circulating strains and egg-based vaccines. QIVc has been associated with fewer symptomatic influenza cases, hospitalizations, and influenza-related complications compared with QIVe, particularly in pediatric and adolescent populations. A recent systematic review evaluating the economic justification for cell-based influenza vaccines in both children and adults confirmed that, across various scenarios and analytical approaches, QIVc consistently demonstrated cost-effectiveness relative to QIVe in diverse global settings (67).

### 4.1 Challenges and considerations

The effectiveness of the influenza vaccine varies from season to season, influenced by multiple factors that affect the match between vaccine strains and circulating strains. This variability can impact the overall cost-effectiveness of vaccination programs. As Kim et al. (38) noted, “More robust models better capturing this diversity in the efficacy of vaccines are needed.”

Another key determinant of cost-effectiveness in vaccination programs is resource allocation. Damm et al. (19) emphasized that cost-effectiveness analyses should incorporate both direct and indirect costs, including patient co-payments and productivity losses. Gregg et al. (47) highlighted substantial indirect cost savings from reduced work absenteeism, demonstrating how vaccination programs can alleviate financial pressures on healthcare systems. Effective resource allocation ensures that vaccination programs remain cost-effective by minimizing wastage and maximizing the efficient use of available resources.

For policymakers in low-resource settings, implementing cost-effective pediatric influenza vaccination programs requires tailored strategies that reflect local epidemiological and economic conditions. Prioritizing high-risk pediatric subgroups, integrating influenza vaccination into existing immunization platforms, and adopting cost-sharing or subsidy mechanisms can enhance program feasibility and sustainability. Strengthening surveillance and improving regional data collection will further support evidence-based decision-making and help optimize resource allocation.

### 4.2 Limitation

This systematic review exclusively examined full-text articles from four electronic databases within a specified timeframe (2013–2024). While our literature search included PubMed, Scopus, Web of Science, and the Cochrane Library to ensure comprehensive coverage, we acknowledge that Embase was not included due to institutional access limitations. Although Embase contains relevant biomedical literature, previous studies have demonstrated substantial overlap with the databases we searched. Nevertheless, the exclusion of Embase may have led to the omission of some studies, which we recognize as a limitation of this review.

Additionally, there is considerable heterogeneity in the designs and methodologies of the included studies, with variations in economic models, assumptions about vaccine effectiveness, and patterns of disease transmission. This heterogeneity presents a significant challenge when attempting to compare results across studies.

To improve comparability and robustness in future research, standardizing key methodological elements—such as time horizons, analytical perspectives (e.g., societal vs. healthcare payer), and cost components—would be beneficial. Greater transparency in reporting and the adoption of common frameworks or guidelines for economic evaluations of influenza vaccination would further facilitate consistent and interpretable findings. In regions with weak surveillance systems, poor data quality and limited availability pose additional barriers to generating reliable and meaningful results, particularly where information on healthcare system variability, economic conditions, and disease burden is lacking. Another challenge is the evolving epidemiology of influenza. The emergence of new strains and changes in healthcare systems are likely to influence cost-effectiveness over time. Furthermore, results are often context-specific, making generalization difficult and replication in other countries or across different age or risk groups challenging. Addressing these limitations is essential to ensure that future reviews yield robust and generalizable conclusions.

## 5 Conclusion

This review demonstrates that vaccinating children against influenza is generally cost-effective, with the greatest value observed in school-aged populations. Quadrivalent vaccines tend to offer greater cost-effectiveness than trivalent formulations. The choice between inactivated and live-attenuated vaccines should be guided by community-specific factors, including population characteristics and risk profiles. Key determinants of cost-effectiveness include vaccine effectiveness, coverage levels, herd immunity, and local economic conditions. Incorporating herd immunity into economic models enhances the accuracy of cost-effectiveness estimates.

## Data Availability

The original contributions presented in the study are included in the article/Supplementary material, further inquiries can be directed to the corresponding author/s.

## References

[1] Nair H Brooks WA Katz M Roca A Berkley JA Madhi SA . Global burden of respiratory infections due to seasonal influenza in young children: a systematic review and meta-analysis. Lancet. (2011) 378:1917–30. doi: 10.1016/S0140-6736(11)61051-9, PMID: 22078723

[2] World Health Organization (WHO). Vaccines against influenza WHO position paper – November 2012. Releve Epidemiol Hebd. (2012) 87:461–76. 23210147

[3] Haugh M Gresset-Bourgeois V Macabeo B Woods A Samson SI. A trivalent, inactivated influenza vaccine (Vaxigrip®): summary of almost 50 years of experience and more than 1.8 billion doses distributed in over 120 countries. Expert Rev Vaccines. (2017) 16:545–64. doi: 10.1080/14760584.2017.1324302, PMID: 28460594

[4] Ambrose CS Levin MJ. The rationale for quadrivalent influenza vaccines. Hum Vaccin Immunother. (2012) 8:81–8. doi: 10.4161/hv.8.1.17623, PMID: 22252006 PMC3350141

[5] Ritzwoller DP Bridges CB Shetterly S Yamasaki K Kolczak M France EK. Effectiveness of the 2003-2004 influenza vaccine among children 6 months to 8 years of age, with 1 vs 2 doses. Pediatrics. (2005) 116:153–9. doi: 10.1542/peds.2005-0049, PMID: 15995046

[6] World Health Organization (WHO) (2023). Influenza (Seasonal). Available online at: https://www.who.int/newsroom/factsheets/detail/influenza(seasonal) (Accessed 14 October 2024)

[7] NHS (2020). The national flu immunisation programme 2020/21. Available online at: https://assets.publishing.service.gov.uk/government/uploads/system/uploads/attachment_data/file/885281/The_national_flu_immunisation_programme_2020_to_2021.pdf (Accessed 23 December 2024)

[8] THL (2023). Influenza vaccine - THL. Finnish Institute for Health and Welfare (THL), Finland. Available online at: https://thl.fi/en/web/infectious-diseases-and-vaccinations/vaccines-a-to-z/influenza-vaccine (Accessed 23 December 2024)

[9] ECDC (2023). Seasonal influenza vaccination recommendations and coverage rates in EU/EEA member states. Available online at: https://www.ecdc.europa.eu/sites/default/files/documents/Seasonal-flu-vacc-recs-coverage-rates-EU-EEA.pdf (Accessed 23 December 2024)

[10] Sosyal Güvenlik Kurumu (SGK) (2024). Sosyal Güvenlik Kurumu. Available online at: https://www.sgk.gov.tr/Duyuru/Detay/Grip-Asilari-2023-01-30-11-39-14 (Accessed 23 December 2024)

[11] Shang M Blanton L Brammer L Olsen SJ Fry AM. Influenza-associated Pediatric deaths in the United States, 2010-2016. Pediatrics. (2018) 141:e20172918. doi: 10.1542/peds.2017-2918, PMID: 29440502

[12] Charu V Viboud C Simonsen L Sturm-Ramirez K Shinjoh M Chowell G . Influenza-related mortality trends in Japanese and American seniors: evidence for the indirect mortality benefits of vaccinating school children. PLoS One. (2011) 6:e26282. doi: 10.1371/journal.pone.0026282, PMID: 22087226 PMC3210121

[13] Villani L D'Ambrosio F Ricciardi R de Waure C Calabrò GE. Seasonal influenza in children: costs for the health system and society in Europe. Influenza Other Respir Viruses. (2022) 16:820–31. doi: 10.1111/irv.12991, PMID: 35429133 PMC9343336

[14] Cohen GM Nettleman MD. Economic impact of influenza vaccination in preschool children. Pediatrics. (2000) 106:973–6. doi: 10.1542/peds.106.5.973, PMID: 11061762

[15] Meltzer MI Neuzil KM Griffin MR Fukuda K. An economic analysis of annual influenza vaccination of children. Vaccine. (2005) 23:1004–14. doi: 10.1016/j.vaccine.2004.07.040, PMID: 15620473

[16] Page MJ McKenzie JE Bossuyt PM Boutron I Hoffmann TC Mulrow CD . The PRISMA 2020 statement: an updated guideline for reporting systematic reviews. BMJ. (2021) 372:n71. doi: 10.1136/bmj.n71, PMID: 33782057 PMC8005924

[17] Baguelin M Camacho A Flasche S Edmunds WJ. Extending the elderly- and risk-group programme of vaccination against seasonal influenza in England and Wales: a cost-effectiveness study. BMC Med. (2015) 13:236. doi: 10.1186/s12916-015-0452-y, PMID: 26459265 PMC4604076

[18] Crépey P Redondo E Díez-Domingo J Ortiz de Lejarazu R Martinón-Torres F Gil de Miguel Á . From trivalent to quadrivalent influenza vaccines: public health and economic burden for different immunization strategies in Spain. PLoS One. (2020) 15:e0233526. doi: 10.1371/journal.pone.0233526, PMID: 32437476 PMC7241783

[19] Damm O Eichner M Rose MA Knuf M Wutzler P Liese JG . Public health impact and cost-effectiveness of intranasal live attenuated influenza vaccination of children in Germany. Eur J Health Econ. (2015) 16:471–88. doi: 10.1007/s10198-014-0586-4, PMID: 24859492 PMC4435640

[20] de Boer PT Backer JA van Hoek AJ Wallinga J. Vaccinating children against influenza: overall cost-effective with potential for undesirable outcomes. BMC Med. (2020) 18:11. doi: 10.1186/s12916-019-1471-x, PMID: 31931789 PMC6958762

[21] de Boer PT Crépey P Pitman RJ Macabeo B Chit A Postma MJ. Cost-effectiveness of quadrivalent versus trivalent influenza vaccine in the United States. Value Health. (2016) 19:964–75. doi: 10.1016/j.jval.2016.05.012, PMID: 27987647

[22] Gerlier L Lamotte M Grenèche S Lenne X Carrat F Weil-Olivier C . Assessment of public health and economic impact of intranasal live-attenuated influenza vaccination of children in France using a dynamic transmission model. Appl Health Econ Health Policy. (2017) 15:261–76. doi: 10.1007/s40258-016-0296-4, PMID: 27943165

[23] Nagy L Heikkinen T Sackeyfio A Pitman R. The clinical impact and cost effectiveness of quadrivalent versus trivalent influenza vaccination in Finland. PharmacoEconomics. (2016) 34:939–51. doi: 10.1007/s40273-016-0430-z, PMID: 27423657 PMC4980401

[24] Pelton SI Mould-Quevedo JF Nguyen VH. Modelling the population-level benefits and cost-effectiveness of cell-based quadrivalent influenza vaccine for children and adolescents aged 6 months to 17 years in the US. Expert Rev Vaccines. (2024) 23:82–7. doi: 10.1080/14760584.2023.2295014, PMID: 38093415

[25] Pitman RJ Nagy LD Sculpher MJ. Cost-effectiveness of childhood influenza vaccination in England and Wales: results from a dynamic transmission model. Vaccine. (2013) 31:927–42. doi: 10.1016/j.vaccine.2012.12.010, PMID: 23246550

[26] Sandmann FG van Leeuwen E Bernard-Stoecklin S Casado I Castilla J Domegan L . Health and economic impact of seasonal influenza mass vaccination strategies in European settings: a mathematical modelling and cost-effectiveness analysis. Vaccine. (2022) 40:1306–15. doi: 10.1016/j.vaccine.2022.01.015, PMID: 35109968 PMC8861572

[27] Scholz SM Weidemann F Damm O Ultsch B Greiner W Wichmann O. Cost-effectiveness of routine childhood vaccination against seasonal influenza in Germany. Value Health. (2021) 24:32–40. doi: 10.1016/j.jval.2020.05.022, PMID: 33431151

[28] Thommes EW Ismaila A Chit A Meier G Bauch CT. Cost-effectiveness evaluation of quadrivalent influenza vaccines for seasonal influenza prevention: a dynamic modeling study of Canada and the United Kingdom. BMC Infect Dis. (2015) 15:465. doi: 10.1186/s12879-015-1193-4, PMID: 26503131 PMC4623926

[29] Thorrington D van Leeuwen E Ramsay M Pebody R Baguelin M. Cost-effectiveness analysis of quadrivalent seasonal influenza vaccines in England. BMC Med. (2017) 15:166. doi: 10.1186/s12916-017-0932-3, PMID: 28882149 PMC5590113

[30] Wenzel NS Atkins KE van Leeuwen E Halloran ME Baguelin M. Cost-effectiveness of live-attenuated influenza vaccination among school-age children. Vaccine. (2021) 39:447–56. doi: 10.1016/j.vaccine.2020.10.007, PMID: 33280855

[31] Bellier L Petitjean A Sarazu T Tresierra J Lopez JG. Cost-effectiveness analysis of switching from a trivalent to a quadrivalent inactivated influenza vaccine in the Peruvian immunisation programme. Vaccine. (2021) 39:4144–52. doi: 10.1016/j.vaccine.2021.05.084, PMID: 34130885

[32] Chi CY Cheng MF Ko K Mould JF Chen CJ Huang YC . Cost-effectiveness analysis of cell-based versus egg-based quadrivalent influenza vaccines in the pediatric population in Taiwan. J Med Virol. (2024) 96:e29279. doi: 10.1002/jmv.29279, PMID: 38196182

[33] Chit A Roiz J Aballea S. An assessment of the expected cost-effectiveness of quadrivalent influenza vaccines in Ontario, Canada using a static model. PLoS One. (2015) 10:e0133606. doi: 10.1371/journal.pone.0133606, PMID: 26222538 PMC4519190

[34] Gong Y Yao X Peng J Ma Y Fang Y Yan K . Cost-effectiveness and health impacts of different influenza vaccination strategies for children in China. American Journal of Preventive Medicine. (2023) 65:155–164. 37037733 10.1016/j.amepre.2023.01.028

[35] Hart RJ Stevenson MD Smith MJ LaJoie AS Cross K. Cost-effectiveness of strategies for offering influenza vaccine in the Pediatric emergency department. JAMA Pediatr. (2018) 172:e173879. doi: 10.1001/jamapediatrics.2017.3879, PMID: 29114729 PMC6583269

[36] Hassan MZ Jubayer Biswas MAA Shirin T Rahman M Chowdhury F Azziz-Baumgartner E . Cost-effectiveness of seasonal influenza vaccination in WHO-defined high-risk populations in Bangladesh. J Glob Health. (2024) 14:04126. doi: 10.7189/jogh.14.04126, PMID: 39024624 PMC11257706

[37] Kim DeLuca E Gebremariam A Rose A Biggerstaff M Meltzer MI Prosser LA. Cost-effectiveness of routine annual influenza vaccination by age and risk status. Vaccine. (2023) 41:4239–48. doi: 10.1016/j.vaccine.2023.04.069, PMID: 37291022

[38] Kim YK Song JY Jang H Kim TH Koo H Varghese L . Cost effectiveness of quadrivalent influenza vaccines compared with trivalent influenza vaccines in young children and older adults in Korea. PharmacoEconomics. (2018) 36:1475–90. doi: 10.1007/s40273-018-0715-5, PMID: 30251078 PMC6244612

[39] Kittikraisak W Suntarattiwong P Ditsungnoen D Pallas SE Abimbola TO Klungthong C . Cost-effectiveness of inactivated seasonal influenza vaccination in a cohort of Thai children ≤60 months of age. PLoS One. (2017) 12:e0183391. doi: 10.1371/journal.pone.0183391, PMID: 28837594 PMC5570265

[40] Naber SK Bruijning-Verhagen PCJL de Hoog MLA van Giessen A. Cost-effectiveness of inactivated influenza vaccination in children with medical risk conditions in the Netherlands. Vaccine. (2020) 38:3387–96. doi: 10.1016/j.vaccine.2020.01.057, PMID: 32115297

[41] Ruiz-Aragón J Gani R Márquez S Alvarez P. Estimated cost-effectiveness and burden of disease associated with quadrivalent cell-based and egg-based influenza vaccines in Spain. Hum Vaccin Immunother. (2020) 16:2238–44. doi: 10.1080/21645515.2020.1712935, PMID: 32040379 PMC7553711

[42] Urueña A Micone P Magneres MC McGovern I Mould-Quevedo J Sarmento TTR . Cost-effectiveness analysis of cell versus egg-based seasonal influenza vaccination in children and adults in Argentina. Vaccines. (2022) 10:1627. doi: 10.3390/vaccines10101627, PMID: 36298493 PMC9612026

[43] Vo TQ Van Hoang M Riewpaiboon A. Modelling the health economic impact of influenza vaccination strategies for high-risk children in Vietnam. J Clin Diagn Res. (2018) 12:LC26–32. doi: 10.7860/jcdr/2018/35722.11702

[44] Wang Q Jin H Yang L Jin H Lin L. Cost-effectiveness of seasonal influenza vaccination of children in China: a modeling analysis. Infect Dis Poverty. (2023) 12:92. doi: 10.1186/s40249-023-01144-6, PMID: 37821942 PMC10566174

[45] Wong C Jiang M You JH. Potential cost-effectiveness of an influenza vaccination program offering microneedle patch for vaccine delivery in children. PLoS One. (2016) 11:e0169030. doi: 10.1371/journal.pone.0169030, PMID: 28006012 PMC5179085

[46] Yoo BK Humiston SG Szilagyi PG Schaffer SJ Long C Kolasa M. Cost effectiveness analysis of elementary school-located vaccination against influenza--results from a randomized controlled trial. Vaccine. (2013) 31:2156–64. doi: 10.1016/j.vaccine.2013.02.052, PMID: 23499607 PMC4583066

[47] Gregg M Blackhouse G Loeb M Goeree R. Economic evaluation of an influenza immunization strategy of healthy children. Int J Technol Assess Health Care. (2014) 30:394–9. doi: 10.1017/S0266462314000397, PMID: 25412647

[48] Bianculli PM Bellier L Mangado IO Pérez CG Mieres G Lazarov L . Switching from trivalent to quadrivalent inactivated influenza vaccines in Uruguay: a cost-effectiveness analysis. Hum Vaccin Immunother. (2022) 18:2050653. doi: 10.1080/21645515.2022.2050653, PMID: 35344679 PMC9225211

[49] Edoka I Kohli-Lynch C Fraser H Hofman K Tempia S McMorrow M . A cost-effectiveness analysis of South Africa's seasonal influenza vaccination programme. Vaccine. (2021) 39:412–22. doi: 10.1016/j.vaccine.2020.11.028, PMID: 33272702 PMC7804372

[50] Yang KC Hung HF Chen MK Chen SL Fann JC Chiu SY . Cost-effectiveness analysis of universal influenza vaccination: application of the susceptible-infectious-complication-recovery model. Int J Infect Dis. (2018) 73:102–8. doi: 10.1016/j.ijid.2018.05.024, PMID: 29906602

[51] Baguelin M Flasche S Camacho A Demiris N Miller E Edmunds WJ. Assessing optimal target populations for influenza vaccination programmes: an evidence synthesis and modelling study. PLoS Med. (2013) 10:e1001527. doi: 10.1371/journal.pmed.1001527, PMID: 24115913 PMC3793005

[52] Medlock J Galvani AP. Optimizing influenza vaccine distribution. Science. (2009) 325:1705–8. doi: 10.1126/science.1175570, PMID: 19696313

[53] Boccalini S Bechini A Moscadelli A Paoli S Schirripa A Bonanni P. Cost-effectiveness of childhood influenza vaccination in Europe: results from a systematic review. Expert Rev Pharmacoecon Outcomes Res. (2021) 21:911–22. doi: 10.1080/14737167.2021.1925110, PMID: 33930994

[54] Basta NE Chao DL Halloran ME Matrajt L Longini IM Jr. Strategies for pandemic and seasonal influenza vaccination of schoolchildren in the United States. Am J Epidemiol. (2009) 170:679–86. doi: 10.1093/aje/kwp237, PMID: 19679750 PMC2737588

[55] Reichert TA Sugaya N Fedson DS Glezen WP Simonsen L Tashiro M. The Japanese experience with vaccinating schoolchildren against influenza. N Engl J Med. (2001) 344:889–96. doi: 10.1056/NEJM200103223441204, PMID: 11259722

[56] Eichner M Schwehm M Eichner L Gerlier L. Direct and indirect effects of influenza vaccination. BMC Infect Dis. (2017) 17:356. doi: 10.1186/s12879-017-2399-4 28441935 PMC5405516

[57] Weycker D Edelsberg J Halloran ME Longini IM Jr Nizam A Ciuryla V . Population-wide benefits of routine vaccination of children against influenza. Vaccine. (2005) 23:1284–93. doi: 10.1016/j.vaccine.2004.08.044, PMID: 15652671

[58] Talbird SE Carrico J La EM Carias C Marshall GS Roberts CS . Current estimates of the impact of routine childhood immunizations in reducing vaccine-preventable diseases in the United States. Open Forum Infect Dis. (2020) 7:S703. doi: 10.1093/ofid/ofaa439.1569 35821599

[59] Pradas-Velasco R Antoñanzas-Villar F Martínez-Zárate MP. Dynamic modelling of infectious diseases: an application to the economic evaluation of influenza vaccination. PharmacoEconomics. (2008) 26:45–56. doi: 10.2165/00019053-200826010-00005 18088158

[60] Loong D Amiri M Saunders H Mishra S Radhakrishnan A Rodrigues M . Systematic review on the cost-effectiveness of seasonal influenza vaccines in older adults. Value Health. (2022) 25:1439–58. doi: 10.1016/j.jval.2022.03.011, PMID: 35659487

[61] Ozawa S Mirelman A Stack ML Walker DG Levine OS. Cost-effectiveness and economic benefits of vaccines in low-and middle-income countries: a systematic review. Vaccine. (2012) 31:96–108. doi: 10.1016/j.vaccine.2012.10.103, PMID: 23142307

[62] Iino H Hashiguchi M Hori S. Estimating the range of incremental cost-effectiveness thresholds for healthcare based on willingness to pay and GDP per capita: A systematic review. PLoS ONE. (2022) 17. doi: 10.1371/journal.pone.0266934 PMC9009631 35421181

[63] de Boer PT van Maanen BM Damm O Ultsch B Dolk FC Crépey P . A systematic review of the health economic consequences of quadrivalent influenza vaccination. Expert Rev Pharmacoecon Outcomes Res. (2017) 17:249–65. doi: 10.1080/14737167.2017.1343145 28613092

[64] Chan K Wong C Choi H. Cost-effectiveness of intranasal live-attenuated influenza vaccine for children: a systematic review. Vaccines. (2022) 10:1466. doi: 10.3390/vaccines10091466, PMID: 36146544 PMC9505322

[65] Luce BR Nichol KL Belshe RB Frick KD Li SX Boscoe A . Cost-effectiveness of live attenuated influenza vaccine versus inactivated influenza vaccine among children aged 24-59 months in the United States. Vaccine. (2008) 26:2841–8. doi: 10.1016/j.vaccine.2008.03.046, PMID: 18462851

[66] Tarride JE Burke N Von Keyserlingk C O'Reilly D Xie F Goeree R. Cost-effectiveness analysis of intranasal live attenuated vaccine (LAIV) versus injectable inactivated influenza vaccine (TIV) for Canadian children and adolescents. Clinicoecon Outcomes Res. (2012) 4:287–98. doi: 10.2147/CEOR.S33444, PMID: 23055756 PMC3468276

[67] Fisman D Giglio N Levin MJ Nguyen VH Pelton SI Postma M . The economic rationale for cell-based influenza vaccines in children and adults: a review of cost-effectiveness analyses. Hum Vaccin Immunother. (2024) 20:2351675. doi: 10.1080/21645515.2024.2351675, PMID: 38835218 PMC11155702

