# Supplementary material for: Optimizing influenza prevention: a systematic review of the cost-effectiveness of pediatric vaccination programs and vaccine types
Source: Front Public Health. 2025 Oct 30;13:1589403. doi: 10.3389/fpubh.2025.1589403 (PMC12611858; doi:10.3389/fpubh.2025.1589403)
Supplement: Supplementary file 1 [file Table_1.docx]

| Supplementary Table 1. **Characteristics of studies included in the systematic review** | | | | | | | | | |
| --- | --- | --- | --- | --- | --- | --- | --- | --- | --- |
| Authors | Year | Setting | Study aim | Type of vaccine | Population | Type of economic evaluation | Perspective | Time horizon | Mathematical method |
| Pitman et al | 2013 | England | To compare the cost-effectiveness of various pediatric influenza vaccination policies in England and Wales. | LAIV+ current practice versus TIV plus current practice | 2-18 years old | Cost-effectiveness | NHS perspective | 200 years | Dynamic  transmission  method |
| Yoo et al | 2013 | USA | To compare cost-effectiveness of school-located vs office-based influenza vaccination in elementary children | LAIV (nasal spray) & TIV (injection) | School‐aged children roughly 5 to 11 years old | Cost-effectiveness | Narrow societal perspective.  Broader societal perspective.  School-district/local health-department | One influenza season (2009–2010) | Static, decision-analytic model |
| Gregg et al | 2014 | Canada | To evaluate the cost-effectiveness of immunizing healthy children against influenza to protect entire communities. | TIV or hepatitis A vaccine | 36 months to 15 years | cost-effectiveness | Societal perspective | 6 months | Static model |
| Chit et al | 2015 | Canada | To assess the expected Cost-Effectiveness of Quadrivalent Influenza Vaccines in Ontario, Canada | IIV3 versus IIV4 | All Ontarians over 6 months of age (Universal Influenza Immunization Program, UIIP) | Cost-utility | Ontario ministry of health (MOH) and societal perspective | One influenza season | Static Model |
| Damm et al | 2015 | Germany | To estimate the epidemiological impact and cost-effectiveness of intranasal live attenuated influenza vaccination of children in Germany. | LAIV versus TIV | 2-17 years | Cost-effectiveness | A societal perspective, narrow third-party payer perspective, broad third-payer perspective | 10 years | Dynamic  transmission  method |
| Thommes et al | 2015 | Canada and UK | To assess the potential vaccine impacts and cost-effectiveness of switching from TIV to QIV in Canada and the UK. | TIV TO QIV for all population in Canada, while in the UK children aged 2-17 years change from LAIV TO QLAIV and those one over 18 from TIV to QIV. | All population in Canada, UK 2-17 years old and >18. | Cost-effectiveness | A third-party payer perspective. | 10 years | Dynamic  Transmission method. |
| Baguelin et al | 2015 | England and Wales | To evaluate the cost-effectiveness of extending the influenza immunization program to low-risk individuals in England and Wales. | LAIV for children 2-16 years and TIV for the others | pre-school children ( 2–4 years), school-aged children ( 5–16 years), adults ( 50–64 years), the elderly ( 65 years and over), and high-risk individuals | Cost-effectiveness | Health service and personal social services perspective | 14 influenza seasons (1995–2009). | Dynamic  transmission  method. |
| Boer et al | 2016 | United State | To estimate the cost-effectiveness of replacing trivalent influenza vaccine (TIV) with quadrivalent influenza vaccine (QIV) in the United States. | TIV versus QIV | 0–23 months, 2 years, 3–4 years, 5–11 years, 12–17 years, 18–49 years, 50–64 years, 65+ years | Cost-effectiveness analysis | Societal and third-party payer (TPP) perspectives | 20 years (2014–2034) | Dynamic  transmission  method. |
| Nagy et al | 2016 | Finland | To assess the cost-effectiveness of switching from TIV to quadrivalent vaccination in Finland. | TIV to Q-LAIV in children and QIV in other ages compared to other scenarios and no vaccination | 2 - <18 years  18 to <65 years  65 years and older: | Cost Effectiveness | Societal perspective and payer perspective | 20 years | Dynamic  transmission  method |
| Wong et al | 2016 | China | To evaluate potential clinical outcomes & direct medical costs of offering a microneedle-patch (MNP) alternative to IM vaccine | IM quadrivalent vs. MNP-delivered quadrivalent influenza vaccine | Children 6 mo–6 y | Cost Effectiveness | Healthcare provider | 1 year | Static decision tree model |
| Gerlier et al | 2017 | France | To estimate the epidemiological and economic impact of extending the French vaccination program to healthy children. | QLAIV/TIV. | children aged 2–17 years  Adult >18 | Cost-effectiveness | All-payer perspectives in addition to a societal perspective | 10 years | Dynamic  transmission  method |
| Kittikraisak et al. | 2017 | Bangkok, Thailand | To evaluate the cost-effectiveness of trivalent inactivated seasonal influenza vaccine (IIV3) versus no vaccination in children aged ≤ 60 months. | IIV3 delivered as half of an adult ready-to-use dose per child | Children ≤ 60 months | Cost-effectiveness | Societal | One year per influenza season, modelled separately for 2012–13, 2013–14, and 2014–15 seasons. | Static decision-tree model |
| Thorrington et al | 2017 | England | To estimate the cost-effectiveness of quadrivalent influenza vaccines in England. | QLAIV for healthy children without contraindications, QIIV for children with contraindications, clinical risk, and elderly individuals, and TIIV as a comparator. | healthy children aged 2–16 years, children with contraindications, clinical risk groups aged under 65 years, and elderly individuals aged 65 years and older | Cost-effectiveness | Healthcare provider perspective | 14 years | Dynamic  transmission  method |
| Hart et al | 2018 | USA | To compare cost-effectiveness of 4 PED-based influenza vaccine strategies (all patients; <5 y; high-risk only; none) | Seasonal inactivated influenza vaccine | Children (<18) visiting PED in season | Cost-effectiveness | Societal | 1 influenza season | Static decision-tree model |
| Kim et al | 2018 | South Korean | To compare the cost-effectiveness of QIV versus TIV in children and older adults in South Korea. | QIV versus TIV | 6-56 months and > = 65 years aged | Cost Effectiveness | Societal perspective | 1 year | Static model |
| Vo et al | 2018 | Vietnam | To evaluate the cost-effectiveness and budget impact of influenza vaccination strategies for high-risk children (<15 years). | TIV | Children aged 0–15 years | Cost-Effectiveness Analysis | Social, Healthcare provider | One influenza season (Jan–Dec 2009) | Static, decision-tree model |
| Ruiz-Aragón et al | 2020 | Spain | To estimate the cost-effectiveness and disease burden associated with quadrivalent cell-based (QIVc) and egg-based (QIVe) influenza vaccines in Spain. | QIVc /QIVe | 9–17, 18–59, and 60–64 years. | Cost-effectiveness analysis | Public payer and societal perspectives | One influenza season (one year) | Static decision-tree model |
| Crépey et al | 2020 | Spain | To estimate the public health and economic impact of switching from TIV to QIV for different immunization strategies in Spain | TIV/ QIV | 0–1, 2–4, 5–14, 15–19, 20–49, 50–64, 65–69, 70–74, and 75+ years. But the ICER calculated as a total and for > 65 | Cost-effectiveness analysis | Payer perspective and societal perspective | One influenza season | Dynamic transmission model |
| De Boer et al | 2020 | Netherland | To assess the cost-effectiveness of an influenza vaccination program for children in the Netherlands. | QLAIV (In addition to the current strategy) | 2–16 years | Cost-effectiveness | a societal perspective. | 20 seasons. | Stochastic dynamic transmission mode |
| Naber S et al | 2020 | Netherlands | To evaluate the cost-effectiveness of IIV immunization in children with medical risk conditions in the Netherlands. | Inactivated influenza vaccine versus no immunization | 0-17 high-risk group children | Cost-effectiveness | Societal perspective | 1 year | Static model |
| Wenzel et al | 2020 | England & Wales | To compare cost-effectiveness of seven alternative LAIV strategies targeting subgroups of 2–16 y children | (LAIV; Fluenz Tetra nasal spray) | Low-risk children aged 2–16 y (stratified into preschool 2–4 y; primary 5–11 y; secondary 12–16 y); status-quo includes high-risk 6 m–65 y and ≥65 y | Cost-effectiveness | NHS | Annual (one-season) simulations repeated over 19 seasons; one-year horizon per season; 3.5% discount rate |  |
| Bellier et al | 2021 | Peru | To assess the cost-effectiveness of switching from TIV to QIV in the Peruvian immunization program. | QIV versus TIV | Children ≤2 years Pregnant women  Individuals ≥60 years  Healthcare workers  Individuals with underlying medical conditions | Cost-effectiveness | Payer and Societal Perspectives | Single influenza season | Static Model |
| Edoka et al | 2021 | South Africa | To assess the cost-effectiveness of South Africa’s seasonal influenza vaccination strategy. | TIV vaccination versus no vaccine | Persons aged ≥ 65 years  Pregnant women, persons living with HIV/AIDS (PLWHA), persons of any age with underlying medical conditions (UMC), and children aged 6-59 months. | cost-effectiveness | Public healthcare provider and societal perspective | Vaccination campaign, which typically runs from approximately March to July. | “CETSIV” tool static model |
| Scholz et al | 2021 | Germany | To examine the cost-effectiveness of extending the recommendation of childhood vaccination against seasonal influenza in Germany. | TV,  iTV,  QV | 2 - 9 years and 2 - 17 years. | Cost-effectiveness | A societal and a third-party payer (TPP) perspective. | 10 non-pandemic seasons, from 2003/2004 to 2013/2014. | A dynamic transmission mode |
| Bianculli et al | 2022 | Uruguay | To evaluate the cost-utility of replacing TIV with QIV in Uruguay. | QIV versus TIV | All ages  children aged ≤4 years and adults aged ≥65 years | cost-effectiveness | Payer and Societal Perspective | 2013 to 2019 seasons  one average season but also long-term complications are taken into account | Static Model |
| Sandmann et al | 2022 | Europe (England, France, Ireland, Navarra, Netherlands, Portugal, Scotland and Spain) | To explore the impact and cost-effectiveness of various vaccination programs targeting the elderly and/or children in eight European settings. | The pediatric population takes TIV or QIV. | All ages  aged ≥65 or ≥55 years.  Children uptoage 16 | Cost-effectiveness | Healthcare provider perspective | The post-pandemic seasons from 2010/11 to 2017/18 (and from 2014/15 for France) | Structured dynamic-transmission model |
| Urueña et al | 2022 | Argentina | To compare cost-effectiveness of quadrivalent cell-based (QIVc) vs egg-based (QIVe) seasonal influenza vaccination | QIVc versus QIVe | 6–23 mo; 2–4 y; 5–14 y; 15–64 y.  Chronic comorbidities for 2–64 y groups) | Cost-effectiveness | Payer and societal | One-year | Static decision-tree model |
| Kim Deluca et al | 2023 | US | To evaluate the cost-effectiveness of routine annual influenza vaccination in the US by age and risk status. | IIV4, RIV4,  HD-IIV4 aIIV4. | 6–23 months  2–4 years  5–11 years  12–17 years  18–49 years  50–64 years  65 years and older | Cost-effectiveness | Societal perspective and health sector perspective | one year | State-transition simulation model |
| Wang et al | 2023 | China | To evaluate the cost-effectiveness of introducing government-funded influenza vaccination to children in China. | TIV | 6 months to 14 years | Cost-effectiveness | Societal Perspective | One influenza season | Static model |
| Gong et al | 2023 | China | To evaluate the economic and health impacts of different influenza vaccines for children in China. | TIV, QIV, LAIV | Six months to 18 years. | Cost-effectiveness | Societal perspective | One year | Static model |
| Hassan et al | 2024 | Bangladesh | To determine the cost-effectiveness of influenza vaccination in high-risk populations in Bangladesh. | NR | Pregnant women, children aged 6–59 months, older adults (≥60 years), healthcare personnel, and adults with chronic medical conditions | Cost-effectiveness | Societal perspective t | One year | static model |
| Chi et al | 2024 | Taiwan | To assess the cost-effectiveness of cell-based versus egg-based quadrivalent influenza vaccines in the pediatric population in Taiwan. | QIVc /QIVe | (6 months - 17 years) | Cost‐effectiveness | Payer and Societal Perspective | one‐season | Static Model |
| Pelton et al | 2024 | US | To evaluate the population-level cost-effectiveness of cell-based quadrivalent influenza vaccine for children and adolescents in the US. | QIVc  QIVe | Children and adolescents aged 6 months to 17 years | Cost‐effectiveness | Payer and Societal Perspective | The duration of typical influenza is October to May | Dynamic transmission model |

1. NHS: National Health Service, LAIV: Trivalent Live Attenuated Vaccine, TIV or TIIV,: Trivalent Inactivated Influenza Vaccine, QIV or QIIV:: Quadrivalent Inactivated Influenza Vaccine, QLAIV: Quadrivalent Live Attenuated Vaccine, TPP: Third-Party Payer, iTV: Improved trivalent vaccine, IIV4: Standard-dose quadrivalent inactivated influenza vaccine, RIV4: recombinant quadrivalent influenza, HD-IIV4: high-dose, MNP: microneedle patch delivery.
