# Supplementary material for: Optimizing influenza prevention: a systematic review of the cost-effectiveness of pediatric vaccination programs and vaccine types
Source: Front Public Health. 2025 Oct 30;13:1589403. doi: 10.3389/fpubh.2025.1589403 (PMC12611858; doi:10.3389/fpubh.2025.1589403)
Supplement: Supplementary file 2 [file Table_2.docx]

## Supplementary Table 2. The Main Vaccination Assumption in Economic Models

| Authors | Vaccine Efficacy/Effectivness | Vaccine coverage | Cost per dose |
| --- | --- | --- | --- |
| Pitman et al. (2013) | - TIV Efficacy: 60% for children (0-18 years) - LIV Efficacy: 80% for children (2-18 years) | - 50% for both vaccines | - LAIV and TIV: £5.81 and Total influenza vaccine cost: £38.60 |
| Yoo et al. (2013) | - NR | - Baseline coverage (pre-intervention, via medical practices) in the study site was 29.5 % of school-aged children. | - Vaccine purchase cost used in the model (weighted TIV/LAIV price): $10.40 per dose |
| Gregg et al. (2014) | - Lower cases of influenza 4.5% in the treatment arm (influenza vaccine) vs 10.6% in the control arm (Hepatitis A). relative protective effect of 59%. | - NR | - Vaccine, iQIV (VAXIGRIP $8.77) - Total influenza vaccination cost $100.39/dose |
| Chit et al (2015) | - A/H1N1: 0.58 (0.457–0.698) - A/H3N2: 0.53 (0.410–0.648) - Matched B: 0.47 (0.267–0.678) - Mismatched B: 0.28 (0.166–0.411) | - NR | - IIV3: $5.5 - IIV4: $7 |
| Damm et al. (2015) | - TIV Efficacy: 11% for children under 2, 59% for children 2-7, 68% for healthy adults, and 58% for at-risk patients. - LAIV Efficacy: 80% initially after vaccination, 56% in second season (no immunization). | - For LAIV up to 50%. | - TIV: €10.64, LAIV: € 20.20 |
| Thommes et al. (2015) | - The study assumes TIV and QIV have equal efficacy against influenza A strains. QIV is expected to be more effective than TIV in preventing influenza B illnesses due to its consistent coverage of both B lineages, addressing lineage mismatch. | - NR | - TIV: £6.39, QIV: £9.94, LAIV and QLAIV: £ 14 |
| Baguelin et al. (2015) | - Efficacy for TIV in well-matched years: 70% (<65 years) and 46% (≥65 years) - Efficacy for TIV in poor-match years: 42% (<65 years) and 28% (≥65 years). - LAIV has similar protection in children to TIV in adults. | - In the years of the study, risk group vaccination plateaued around 20% in children (aged 1–14 years) and just below 50% in adults. - Coverage of the extended program: 50% for low-risk groups. | - TIV and LAIV: £ 6.21 - Overall estimate of £15.85 per dose of vaccine |
| De Boer et al. (2016) | - Base case values NR, but the scenario analysis explored ±20% of base-case efficacy, and cross-protection against opposite B lineage (range 40%–95% of matched vaccine efficacy). | - ±20% of base-case coverage. | - Incremental vaccine price of QIV over TIV: $7.63 |
| Nagy et al. (2016) | - Inactivated Vaccine Efficacy (QIV/TIV): - 0 to<18 years: 48% (95% CI: 31–61) - 18 to<65 years: 59% (95% CI: 50–66) - ≥65 years: 50% (95% CI: 39–59) - Live-Attenuated Vaccine Efficacy (Q-LAIV): - 0 to<18 years: 80% (95% CI: 70–87) | - The same Finland vaccine coverage in the 2012–2013 seasons by age groups for all scenarios. | - Price consistency: TIV = QIV and LAIV = Q-LAIV |
| Wong et al. (2016) | - VE (IM): 63 % (95 %CI 52–72 %) | - IM acceptance: 28.4 %MNP increases uptake ×1.33 | - IM: $16.7  MNP: assumed 1.0× (range 1.0–2.0×) |
| Gerlieret al. (2017) | - TIV: 11% (1 year); 59% (2–17 years); 68% (≥18, no risk); 58% (≥18, at-risk) - QLAIV: 80% (2–17 years) | - Vaccination coverage with TIV in at-risk/elderly individuals (current strategy) or gradually extending the vaccination to healthy children (aged 2–17 years) with QLAIV from current uptake up to 50% (evaluated strategy). | - TIV: £6,14, QLAIV: £ 30.37 |
| Kittikraisak et al. (2017) | - 2012 season: 64% - 2013 season: 64% - 2014 season: 24% | - 2012 season: 29% - 2013 season: 31% - 2014 season: 29% | - Purchase, storage, distribution per adult dose: $ 3.64 (2012), 3.68 (2013), 3.48 (2014) - Promotion, administration & supervision per dose: $ 0.85 (2012), 0.86 (2013), 0.77 (2014). - Children received one half-dose each; the other half discarded |
| Thorrington et al. (2017) | - TIV and QIV against influenza A: Equivalent - Efficacy against influenza B: TIV and LAIV: 42% (<65 years) and 28% (>64 years) - QIV and QLAIV: 70% (<65 years) and 46% (>64 years). | - Low risk< 5 years: 33.7% - Low-risk 5–16 years: 54.9% - Clinical risk < 6 months–64 years: 45.1% - ≥65years: 71.0% | - NR |
| Hart et al. (2018) | - VE 0–4 y: 0.53 (0.15–0.90) - VE ≥5 y: 0.593 (0.23–0.69) | - Baseline vaccination 0–4 y: 0.62 (0.41–0.72) - ≥5 y: 0.45 (0.20–0.70) - ED acceptance: 0.55 (0.25–0.75) | - $19.05 (6.93–23.70) |
| Kim et al. (2018) | - In a narrow definition, QIV is expected to prevent nearly 16,000 medically attended influenza cases compared to TIV's broad definition, QIV is projected to prevent over 190,000 influenza cases. - QIV is expected to prevent nearly 8000 cases of complications under the narrow definition - Under the broad definition, QIV is projected to prevent over 37,000 complications QIV is expected to prevent over 230 deaths under the narrow definition - Under the broad definition, QIV is projected to prevent 270 deaths | - The influenza vaccine coverage in children is assumed to be the same as that in older adults, which is 82%. | - KRW 7510 for TIV (based on the 2016 NIP procurement price) and KRW 11,265 for QIV (assuming a 50% premium due to the inclusion of an additional B lineage). |
| Vo et al. (2018) | - Base-case efficacy: 60% (range examined 0–100% strain match). | - Base-case vaccination coverage: 97.2%. | - TIV per dose: $ 10.23 |
| Ruiz-Aragón et al. (2020) | - QIVc is 26.8% more effective than QIVe against A/H3N2, as calculated from published observational data (Boikos et al.). | - 9–17 yrs: 32% - 18–59 yrs: 17% - 60–64 yrs: 22% | - QIVc: €7.50 - QIVe: €6.00 |
| Crépey et al. (2020) | - 0–0.5 years: 0% (no efficacy) - 0.5–5 years:A/H1N1 & A/H3N2: 50.85%, B Victoria & B Yamagata: 61.02% - 5–10 years:A/H1N1 & A/H3N2: 47.3%, B Victoria & B Yamagata: 56.76% - 10–15 years:A/H1N1 & A/H3N2: 41%, B Victoria & B Yamagata: 49.2% - 15–20 years:A/H1N1 & A/H3N2: 41%,B Victoria & B Yamagata: 49.2% - 20–40 years:A/H1N1 & A/H3N2: 41.65%, B Victoria & B Yamagata: 49.98% - 40–60 years:A/H1N1 & A/H3N2: 66.65%, B Victoria & B Yamagata: 79.98% - 60–100 years:A/H1N1 & A/H3N2: 50%, B Victoria & B Yamagata: 60% | - 0–4 years: 1.68% - 5–14 years: 1.68% - 15–44 years: 5.22% - 45–64 years: 15.67% - 65+ years: 58.16% | **TIV :** €2.98 per dose  **QIV:** €7.00 per dose |
| De Boer et al. (2020) | - Effectiveness for TIV and QLAIV against circulating influenza: 45%. - The duration of protection through Q-LAIV is assumed to be 1 year | - 50% | - QLAIV and TIV: €3.59 |
| Naber et al. (2020) | - The study estimates that IIV prevents an average of 1.59 influenza-related hospitalizations and 0.02 deaths per 1,000 children with medical risk conditions. | - NR | - Total costs per vaccination were increased by 14% to €29.20 |
| Wenzel et al. (2020) | - Well-matched years: VE 70% general / 42% high-risk; Poorly matched: VE 40% / 28% high-risk | - Targeted cohorts at 30%, 55%, 70% (baseline empirical for others) | - GP delivery: £19.66; Pharmacy: £17.29; School-delivery: £20.14 |
| Bellier et al. (2021) | - NR | - For children 1 year old the coverage rate is 52.44% - For individuals 18 years and older to 60 years and older, the coverage rates range from 7.18% to 18.55% | - TIV: The average cost of vaccination with TIV was reported as follows: - Pediatric vaccine (for infants under 1 year of age): $1.33 - Adult vaccine: $3.50 - QIV: The average cost of vaccination with QIV was reported as follows: - Pediatric vaccine (for infants under 1 year of age): $2.57 - Adult vaccine: $6.00 |
| Edoka et al. (2021) | TIV efficacy has been shown to be higher in school-age children compared to children aged 6–59 months, which may increase the benefits of vaccinating this sub-population. | - For children aged 6–59 months, data from the 2017 campaign is used due to their exclusion from the 2018 program. - % NR | - Different costs for each population. - For children 6-59 months 6.07$ (2.53-10.02) |
| Scholz et al. (2021) | Reductions in hospitalizations and deaths are 87.2% and 90.5% for the total population and 79.9% and 80.4% for 2- to 9-year-olds, respectively. | - 40% vaccine uptake was assumed for QIV among children aged 2 to 9 years. | - QIV price of €15.97 (minimum €13.82; maximum €24.27) per - Dose and an additional €7.37 for vaccine administration |
| Bianculli et al. (2022) | - QIV efficacy for influenza B was comparable to matched TIV, with 67% cross-protection by TIV against the mismatched B strain. - QIV instead of TIV in Uruguay might prevent 740 influenza cases, 500 GP consultations, 15 hospitalizations, 3 fatalities, and 300 workdays with the same vaccination coverage during an average influenza season. | - Children ≤4 years: 23% - 5–19 years, 20–49 years, 50–64 years who considered as high risk: 10.2% - Adults ≥65 years: 29.3% | - TIV: $2.65 - QIV: $ 5.14 |
| Sandmann et al. (2022) | - Data for the base case scenario came from the ECDC for the seasons 2010/11 to 2016/17, and from another study for the 2017/18 season, but the exact efficacy NR. | - Uptake levels of 10%, 25%, 50%, and 75%were considered. | - Vaccine pricing varied by country and type. They calculated the iTV price using each setting's highest influenza vaccination price. |
| Urueña et al. (2022) | - QIVe absolute VE vs. A strains: 59–61 % - QIVe vs. B strains: 66–77 % - rVE(QIVc vs QIVe): – Children 6 mo–14 y: 8.1 % (0.1–15.4 %) - Adults 15–64 y: 11.4 % (5.8–16.7 %) | - 74.6% (6–23 mo), 83.0% (2–4 y), 49.7% (5–14 y), 49.7% (15–64 y). | - QIVc: $ 7.52 ; QIVe: $ 6.27 |
| Kim Deluca et al. (2023) | - NR for children | - NR | - 6 months-17 years: $16.88 - ≥18 years: $16.75 |
| Wang et al. (2023) | - The TIV efficiency estimates for the general population were used to estimate effectiveness for children. - % of efficacy NR | - Low vaccination coverage among children, estimated to be 25%. - The assumed increase in vaccine coverage estimated to be 40%. | - The prices of the child and adult influenza vaccines were estimated to be 4.56 $ and 6.66 $, respectively |
| Gong et al. (2023) | - Children aged 6 months to 3 years showed 45% efficacy with one dosage and 57% with two doses of TIV. - For children aged 3–5, TIV efficacy was 47% - 5–18 years old vaccine efficacy was 41%. - The relative efficiency of QIV was assumed to be 1.27 times TIV for children aged 3–5 and 1.26 times TIV for those aged 5–18. LAIV efficacy for 3–to 18-year-olds was 62.5%. | - children aged 6 months to 5 years: coverage rate 28.4% - 5 to 18 years: Coverage rate 25.1% | - Cost per Dose: - TIV Cost per Dose: For children aged 6 months to 3 years TIV was $4.37 for a 0.25 mL dose and $7.75 for a 0.5 mL dose. - For children aged 3 to 18 years, the cost per dose was $7.75 for a 0.5 mL dose. - QIV Cost per Dose: The cost per dose of QIV was $18.29. - LAIV Cost per Dose: The cost per dose of LAIV was $46.20. |
| Hassan et al. (2024) | - Vaccine effectivness data adapted from WHO’s pooled estimates for LMICs for the seasonal influenza vaccines in each risk group. | - Children under five years of age.   Coverage rates from (2% -72%). | - Children under five years: $8.84. |
| Chi et al. (2024) | - The relative vaccine effectiveness (rVE) of QIVc versus QIVe for the 4–17years age group was assumed to be 8.1%. - The rVE remained the same for the 6 months to 3 years age group | - The full vaccination rates for the age groups 6 months to 2 years, 3–6 years, and 7–17years were assumed to be 44.4%, 37.4%, and 74.80%, respectively | - The QIVc unit price was assumed to be 25% higher than that of QIVe. |
| Pelton et al. (2024) | - The study assumes a relative vaccine effectiveness (rVE) of 8.1% and 11.4% for QIVc vs QIVe in children and adults, respectively. | - NR | - NR |

LAIV: Trivalent Live Attenuated Vaccine, TIV: Trivalent Inactivated Influenza Vaccine, QIV: Quadrivalent Inactivated Influenza Vaccine, QLAIV: Quadrivalent Live Attenuated Vaccine, TPP: Third-Party Payer, KRW: South Korean won, NR: Not Reported, ECDC: European Centre for Disease Prevention and Control, iTV: Improved trivalent, WHO: World health organization, LMICs: low and middle income countries. VE: Vaccine Effectiveness, IIV3: Trivalent inactivated influenza vaccine, IIV4: Quadrivalent Inactivated Influenza vaccine, H1N1: **Influenza A virus subtype,** H3N2: **Influenza A virus subtype,** MNP: Microneedle patch
