# Supplementary material for: Optimizing influenza prevention: a systematic review of the cost-effectiveness of pediatric vaccination programs and vaccine types
Source: Front Public Health. 2025 Oct 30;13:1589403. doi: 10.3389/fpubh.2025.1589403 (PMC12611858; doi:10.3389/fpubh.2025.1589403)
Supplement: Supplementary file 3 [file Table_3.docx]

## Supplementary Table 3: The Findings and Economic Data of the Research Included in the Review

| Authors | Driver of costs | Clinical Outcomes | ICER | Sensitivity analysis | Conclusion |
| --- | --- | --- | --- | --- | --- |
| Pitman et al. (2013) | Direct costs: GP consultations, drug prescriptions, hospitalizations | QALYs | ICER (£251 per QALY gained) for vaccinating 2–18-year-olds with a 50% annual uptake rate compared to current practice. | A probabilistic sensitivity analysis, Univariate sensitivity analysis. Extreme Value Analysis, Direct Effects Analysis. | Vaccinating children aged 2-18 in England and Wales is a highly cost-effective strategy. |
| Gregg et al. (2014) | Direct costs: Influenza vaccination costs follow-up costs (doctor, ER, hospital visits), and hospital admission per day. Antimicrobial costs,  Indirect cost: value of lost work/school day | Incidence of influenza | ICER was $164.12 ($28.38, $2767.75) per case of influenza averted. | One-way and multi-way sensitivity analysis. | Vaccinating healthy children against influenza can be more costly but effective in preventing cases within communities. |
| Chit et al (2015) | MOH perspective: Direct costs of vaccine and vaccine administration cost, cost of management influenza. Societal perspective includes the indirect cost of productivity losses for the working population in Ontario. | Influenza cases averted.  Decrease in outpatient visit, hospitalization, Deaths.  QALY | <4y: MOH $112,274, Societal $112,017. 5–19y: MOH $174,525, Societal $167,856. 20–49y: MOH $303,851, Societal $76,351 50–64y: MOH $217,878, Societal $146,192. 65+: MOH $36,034, Societal $32,864.  For all Ages: MOH \94,248, Societal $62,792 | Univariate and Multivariate statistical uncertainty analyses. | IIV4 remains cost-effective up to a 53% price premium over IIV3. The data show that while IIV4 reduces influenza cases and GP visits in children, the cost-effectiveness is more favorable in older adults. |
| Damm et al. (2015) | Direct costs: vaccination costs, treatment of adverse events, treatment of influenza, Treatment of AOM, and treatment of CAP.  Indirect cost workdays lost | The number of prevented cases of symptomatic influenza, AOM,  CAP.  The number of prevented hospitalizations due to pneumonia.  The number of prevented antibiotic prescriptions.  The number of prevented deaths due to pneumonia. | From a TPP perspective, routine childhood LAIV vaccination had a discounted ICER of €2,265 per QALY gained compared to the existing approach of vaccinating risk groups with TIV.  For TPP perspective, the ICER was €1,228 per QALY gained. | One-way sensitivity analyses,  Two-way sensitivity analyses  Probabilistic sensitivity analyses. | Intranasal live attenuated influenza vaccination of children in Germany could lead to substantial reductions in influenza-associated disease at a reasonable cost. |
| Thommes et al.(2015) | Direct costs: GP visits, ER visits, hospitalizations. | GP visit, ER visits hospitalization and death.  QALYs | Discounted ICER of $7,961 and £7,989/£7,234 per QALY gained are estimated for Canada and the UK (UK1/UK2) respectively | Univariate, multivariate sensitivity analyses  A probabilistic sensitivity analysis | Switching from TIV to QIV in Canada and the UK would likely be cost-effective and further reduce the burden of influenza. |
| Baguelin et al. (2015) | Direct costs: vaccination costs and healthcare costs (GP consultations and hospitalizations. | Number of Cases, Hospitalizations, and Deaths.  QALYs | The ICER for extending the program to children aged 2–16 years is estimated to be £1,949 per QALY.  The ICER for extending the program to all low-risk individuals (2–64 years) is estimated to be £5,046 per QALY. | Probabilistic sensitivity analysis | Extending the influenza immunization program to low-risk individuals, especially children, in England and Wales is likely to be cost-effective. |
| De Boer et al. (2016) | For third-party payers: only direct cost (Vaccination costs, Outpatient visit costs, Hospitalization costs, Costs of influenza-related deaths). Societal perspective, which also accounts for out-of-pocket-paid over-the-counter medication and indirect costs due to productivity losses. | Decrease the number of influenza B cases. Decrease outpatient visits, hospitalization and deaths. | $31,385/QALY gained (TPP perspective). $27,411/QALY gained (societal perspective). $46,477 per life-year gained (TPP). $40,591 per life-year gained (societal | Deterministic sensitivity analysis.  Probabilistic sensitivity analysis.  Scenario analysis. | Over 20 years, shifting to QIV will decrease the number of influenza B cases by 16.0 million (27.2%), avoid 6.1 million outpatient visits, 137,645 hospitalizations, and 16,199 deaths. (In all the population) |
| Nagy et al. (2016) | Direct costs: direct medical costs, direct travel costs.  Indirect cost: indirect productivity costs | Number of symptomatic infections, primary care consultations, hospitalizations, and deaths averted. | From payer Perspective:  QIV/Q-LAIV (100%): Total costs were €616 million, with 86 thousand QALYs lots. The ICER compared to no vaccination was €5734 per QALY gained. | A univariate sensitivity analysis  Dosage Price Assumptions  Mixing Matrix Structure | Quadrivalent vaccination in Finland is expected to be highly cost-effective, reducing the burden of influenza-related disease. |
| Wong et al. (2016) | Vaccine acquisition Adverse-event management, OTC medications (self-treatment), Outpatient visits. Hospital & ICU stays | Infection rate, hospitalizations, mortality. | 27 200 $/QALY | One-way, three-way, and probabilistic sensitivity analysis | Offering MNP to IM-decliners increases uptake, reduces infections, hospitalizations, mortality, and is cost-effective under thresholds; preference depends on WTP, illness duration, and MNP cost. |
| Gerlier et al. (2017) | Direct costs: vaccination costs, influenza treatment costs including physician consultations, medications, and hospitalizations. Indirect costs: the costs of productivity losses. | Reduction of influenza-related events, and mortality. | The ICER was €18,001 /life-year gained. | Univariate Sensitivity Analysis and  A probabilistic sensitivity analysis, | Extending vaccination to healthy children in France with QLAIV is cost-effective and beneficial to public health and economics. |
| Kittikraisak et al. (2017) | Direct medical, direct non-medical, and indirect/caregiver productivity costs | Reduction in influenza cases, hospitalization. QALY | 2012: ICER US24450/QALY (not cost−effective)  2013:US 554/QALY (highly cost-effective)  2014: US16200/QALY (cost−effective)  Under WHO thresholds (Thailand GDP percapita US16200/QALY | Univariate sensitivity analyses. Multivariate sensitivity. analyses Scenario analyses. | Vaccinating children ≤ 60 months with trivalent inactivated influenza vaccine (IIV3) can be economically favorable From a societal perspective—being cost‐effective or highly cost‐effective in most seasons studied—although the introductory year (2012) fell above the willingness-to-pay threshold. |
| Thorrington et al. (2017) | Direct costs: the incremental cost of the quadrivalent vaccines over the trivalent vaccines, the cost of healthcare resources such as GP consultations and inpatient admissions, and the reimbursement costs for healthcare providers administering the vaccines. | Reduction in ILI cases, GP consultations, hospitalizations, and the overall disease burden caused by influenza B strains. | The ICER results indicate that the pediatric program is cost-effective with an increased cost of £6.36 per dose for QLAIV. If the WTP threshold is £20,000 / QALY | A deterministic sensitivity analysis. | Quadrivalent influenza vaccines in England are cost-effective for specific target groups. |
| Hart et al. (2018) | Both direct medical costs (vaccine acquisition and administration, outpatient/ED visits, hospitalizations, complications, adverse‐event management) and indirect costs (caregiver time and lost wages). | Reduction in influenza cases, hospitalizations, adverse effect and QALY. | Cost per case averted: $114.45  Incremental cost per additional case vs no vaccination: $1 030.11  Cost per QALY gained: $55 258 | One-way, three-way, and probabilistic sensitivity analysis | Routine PED-based influenza vaccination of all eligible children is the most cost-effective strategy with net societal benefit. |
| Kim et al. (2018) | Direct costs: vaccine costs, medical costs to the treatment of influenza cases and its complications, including hospitalization, outpatient treatment, and antiviral medication. | Prevention of influenza cases, complications, and deaths. | Narrow Definition of Influenza, TIV:  68,015.15 KRW /QALY. QIV:  87,816.16 KRW /QALY  Broad Definition of Influenza  TIV:  52,806.85 KRW/ QALY  QIV:  53,937.30 KRW/ QALY | A univariate analysis, B-Mismatch Scenarios, and A probabilistic sensitivity analysis  was performed using a Monte Carlo simulation. | QIV is cost-saving in older adults in South Korea but not cost-effective in young children under certain definitions of influenza. |
| Vo et al. (2018) | Vaccine acquisition. Administration Outpatient service Direct non-medical (transport, meals) Indirect (caregiver productivity loss) Influenza treatment (hospital, OPD, pharmacy) | QALY | High-risk children ICERs $25.31 per QALY (societal perspective) and US $31.03 per QALY (health-care provider perspective) | One-way (deterministic) sensitivity analysis  Probabilistic sensitivity analysis | Influenza vaccination of high-risk children are cost effective in both perspectives |
| Ruiz-Aragón et al. (2020) | Direct and indirect costs. Vaccine cost, healthcare resource use (visits, hospitalizations), productivity loss | Symptomatic cases, primary care visits, emergency visits, hospitalizations, deaths, QALYs gained | €12,852 per QALY gained (payer); QIVc dominant (societal) | Deterministic and probabilistic | QIVc is cost-effective vs QIVe for high-risk 9–64 yrs; cost-saving from societal perspective |
| Crépey et al. (2020) | Direct and indirect costs (Vaccine acquisition, administration, outpatient visits, hospitalizations, productivity losses) | Number of influenza B cases, decrease outpatient visits, hospitalizations, and deaths avoided | QIV vs. TIV: €7,007 per QALY | Extensive deterministic and probabilistic sensitivity | Switching from TIV to QIV in Spain would significantly reduce the burden of influenza B, leading to fewer cases, outpatient visits, hospitalizations, and deaths, especially in the elderly. For children exactly NR. |
| De Boer et al. (2020) | Direct Costs: vaccination costs, direct healthcare costs (GP visits, including prescribed medication and referrals to the specialist, and hospitalization costs), patient costs (over-the-counter medication and travel)  Indirect cost: Productivity losses | Number of symptomatic cases,  hospitalization,  death.  QALYS | The childhood vaccination program is estimated to have an average ICER of €3944/ QALY gained simulations. The childhood vaccination program is not estimated to be cost-effective for the target-group itself with an average ICER of €57,054 per QALY gained. | Probabilistic sensitivity analysis. Univariate Sensitivity Analysis | Childhood influenza vaccination in the Netherlands is cost-effective for the general population but not for the target group of children themselves. |
| Naber et al. (2020) | Direct costs: vaccine cost, hospitalization cost.  Indirect cost: productivity losses for work absenteeism among parents. | Reduction in hospitalization, and death. | The ICER for IIV was €50,297/QALY. | Probabilistic sensitivity analysis  One-way sensitivity analysis | Annual IIV immunization for children with medical risk conditions in the Netherlands is unlikely to be cost-effective if it only reduces severe outcomes. |
| Wenzel et al. (2020) | Vaccine acquisition & delivery (purchase, administration, waste disposal, reporting); NHS healthcare costs (GP consultations, hospitalisations | QALYs | V5–11 y (primary school only): £ 639/QALY. V5–16 y (primary + secondary): £ 1 972/QALY. V2–4 y (preschool only): £ 2 054/QALY. V12–16 y (secondary only): £ 2 693/QALY. V2–11 y (preschool + primary): £ 2 761/QALY. V2–16 y (all 2–16 y): £ 2 909/QALY | One-way sensitivity, strain-specific scenario analysis and probabilistic sensitivity analysis | Vaccinating primary school children (5–11 years) with LAIV via school-based delivery is the most cost‐effective strategy (ICER £639/QALY) with a 100 % probability of cost‐effectiveness at the £20 000/QALY threshold, and extending coverage to secondary school children (12–16 years) further increases net health benefits at modest additional cost. |
| Bellier et al. (2021) | Direct costs: vaccine costs, direct Medical Costs: Costs per consultation, hospitalization, and medication.  Indirect Costs: productivity losses | Number of cases prevented  QALYs | payer perspective: ICER was $ 16.649 Cost/QALY from the Societal perspective:  ICER was 13.576 from | Scenario analysis  Deterministic sensitivity analysis Probabilistic Sensitivity Analysis | Introducing QIV in Peru is cost-effective, especially in younger children and the elderly. |
| Edoka et al. (2021) | Direct cost: direct medical and nonmedical costs vaccination program costs, costs associated with outpatient visits and hospitalizations, and the burden of influenza-associated illness.  Indirect costs: productivity loss | QALY | Children aged 6–59 months: $7,500/QALY | Deterministic Probabilistic sensitivity analysis | South Africa’s seasonal influenza vaccination strategy is cost-effective for all vulnerable populations except for young children. |
| Scholz et al. (2021) | Direct costs: outpatient costs, hospitalization costs, vaccination costs, indirect cost Indirect costs: productivity loss, premature mortality | Number of symptomatic cases, consultations, hospitalizations and death. | Societal perspective: vaccinating 2- to 9-year-olds was cost-saving, with a benefit-cost ratio of 1.66.  TPP perspective: the ICER was €998/QALY | Deterministic Probabilistic sensitivity analysis. | Childhood vaccination against seasonal influenza in Germany is cost-saving from a societal perspective and highly cost-effective from a TPP perspective. |
| Bianculli et al. (2022) | Direct costs: treating, managing, and caring for influenza patients’ Indirect costs: productivity loss payers perspective: only direct costs  Society perspective: direct and indirect costs | Number of influenza cases, GP visits, hospitalizations, and fatalities.  QALYs | payer and societal perspectives: ICER for all age groups including children was $18,000 / QALY and around $12,000/QALY for adults ≥65 years of age. | Deterministic Probabilistic sensitivity analysis.  Montecarlo simulation. | Switching to QIV in Uruguay is expected to be cost-effective, particularly for older adults. |
| Sandmann et al. (2022) | Direct cost: medical costs, vaccine costs, administration costs, outpatient consultations, hospitalizations. | Number of ILI cases, hospitalizations, and excess deaths due to influenza.  QALYs | Not clearly defined | Probabilistic sensitivity analysis with Montecarlo sampling | A combination of improved vaccines for the elderly with universal paediatric vaccination programs is supported across European settings. |
| Urueña et al. (2022) | Vaccine acquisition; direct medical (GP visits, complications, hospitalizations); indirect (absenteeism, premature death) | Reduction in GP visits, complications, hospitalizations, deaths, QALY. | Payer perspective: 12,214 $ per QALY gained• Societal perspective: cost‐saving (−1.55 M $ for +282 QALYs) | One‐way deterministic sensitivity analysis  Probabilistic sensitivity analysis | QIVc is more effective and—under most scenarios—cost-effective vs. QIVe for high-risk < 65 y in Argentina; from a societal viewpoint it dominates (cost-saving), with greater benefits in high egg-adaptation seasons. |
| Kim Deluca et al. (2023) | Direct costs: direct medical costs, direct non-medical costs, and opportunity costs associated with vaccination, such as the cost of the vaccine dose itself, administration costs, and the time cost for vaccination. | The number of influenza episodes prevented, hospitalizations prevented, deaths prevented, adverse events incurred. QALYs | ICERs of $ 32,000/QALY for young children aged 2–4 years for non-high-risk subgroups.  ICERs from $1,500/QALY for children aged 2–4 to $40,000/QALY for teenagers aged 12–17 for high-risk group. | One-way sensitivity analysis probabilistic sensitivity analysis and threshold Analyses: | Routine annual influenza vaccination in the US is cost-effective for all age and risk groups except for non-high-risk adults 18-49 years. So its cost effective in low and high risk pediatric groups. |
| Pelton et al. (2023) | Direct costs: outpatient consultations, hospitalizations, vaccine costs, and administration costs.  Indirect costs: productivity losses | QALYs | High Incidence Season:  Payer Perspective: The ICER was -$8,100 /QALY.  Societal Perspective: The ICER was -$15,015/QALY.  Low Incidence Season:  Payer Perspective: The ICER was -$16,427/ QALY.  Societal Perspective: The ICER was -$22,669 /QALY. | Probabilistic sensitivity analysis | Using QIVc instead of QIVe in children and adolescents in the US would reduce the disease burden and be cost-saving. |
| Wang et al. (2023) | Direct costs: vaccine costs, administrative costs, parents time costs, adverse event costs, healthcare costs, Indirect costs | Number of symptomatic cases, hospitalizations, and deaths due to influenza.  QALYs | The fully funded policy ICER: $7964 / QALY gained in 13 of the 31 PLADs. | Deterministic  sensitivity analyses Probabilistic sensitivity  analyses | A government-funded influenza vaccination program for children in China is cost-effective nationally and in many provinces.. |
| Gong et al. (2023) | Direct Costs: costs of inpatient care (ICU and non-ICU), outpatient care, and self-treatment, costs of vaccine and vaccine administration and other non-medical expenses.  Indirect Costs: productivity losses | Numbers of outpatient and inpatient cases, and deaths avoided.  QALYs | For children aged 6 months to 3 years: the ICER for TIV compared to no vaccination was $1,102.5/QALY.  For children aged 3 to 18 years, the ICER for QIV compared to TIV was $32,948.5/QALY.  For children aged 3 to 18 years, the ICER for LAIV compared to QIV was $123,983.8/QALY. | Deterministic sensitivity analyses  Probabilistic sensitivity analyses | Trivalent inactivated vaccine is cost-effective compared with no vaccination in children in China, and QIV appears to be the most cost-effective among three vaccination strategies for older children. |
| Hassan et al. (2024) | Direct cost: medical resource utilization Costs (outpatient care, inpatient care), non-medical resource, utilization costs: food, lodging, transportation. Indirect Costs: productivity Loss | QALYs | Children under five years: $ 4/ QALY gained | Multivariable Probabilistic Sensitivity Analysis, Tornado Analysis | Influenza vaccination for high-risk populations in Bangladesh is either cost-saving or cost-effective. One high-risk group is children under five years |
| Chi et al. (2024) | Direct costs: medical cost and societal cost (transportation per visit to medical facilities  Indirect costs: Productivity loss (parents/caregivers) | Influenza cases –Cases with complications ER visits Hospitalizations Influenza‐related death  QALYs | QIVc had ICER threshold of $ 99 177/QALY. Compared to QIVe, annually.  Payer and Societal perspective: ICERs were $ 68298/QALY and $40 085/QALY, respectively. The payer's ICER was $ 45 782/QALY and the society's $ 17 489/QALY in the high egg adaptations scenario. | A deterministic sensitivity analysis (DSA)  Probabilistic sensitivity | Switching from QIVe to QIVc for children in Taiwan is predicted to reduce the disease burden and be cost-effective. |

GP: General practitioner, ER: Emergency room, LAIV: Trivalent Live Attenuated Vaccine, TIV: Trivalent Inactivated Influenza Vaccine, QIV: Quadrivalent Inactivated Influenza Vaccine, QLAIV: Quadrivalent Live Attenuated Vaccine, TPP: Third-Party Payer, WTP: Weighted Average Treatment Cost, TPP: Third-Party Payer. AOM: acute otitis media, CAP: community-acquired pneumonia, QALYS: Quality-adjusted life years, ILI: influenza-like illness, ICER incremental cost-effectiveness ratio, QIVc: Cell-based Quadrivalent Influenza Vaccine (Surface Antigen, Inactivated), QIVe: Egg-Based Quadrivalent Influenza Vaccine, PLADs: Provincial-level Administrative Divisions
